# Novel Insights into the Pathogenesis of Inflammatory Bowel Diseases

**DOI:** 10.3390/biomedicines13020305

**Published:** 2025-01-26

**Authors:** Valentin Calvez, Pierluigi Puca, Federica Di Vincenzo, Angelo Del Gaudio, Bianca Bartocci, Marco Murgiano, Jacopo Iaccarino, Erfan Parand, Daniele Napolitano, Daniela Pugliese, Antonio Gasbarrini, Franco Scaldaferri

**Affiliations:** 1 IBD Unit, UOC CEMAD Medicina Interna e Gastroenterologia, Centro Malattie dell’Apparato Digerente, Dipartimento di Scienze Mediche e Chirurgiche Addominali ed Endocrino Metaboliche, Fondazione Policlinico Universitario Agostino Gemelli IRCCS, 00168 Rome, Italy; valentino.calvez@gmail.com (V.C.); pierluigi.puca@unicatt.it (P.P.); danielenapolitano1977@gmail.com (D.N.); daniela.pugliese@policlinicogemelli.it (D.P.); 2 Dipartimento di Medicina e Chirurgia Traslazionale, Università Cattolica del Sacro Cuore, 00168 Rome, Italy; federica.divincenzo30@gmail.com (F.D.V.); delgaudioangelo@gmail.com (A.D.G.); biancab97@hotmail.it (B.B.); marcomurgiano1@gmail.com (M.M.); ja.iaccarino@gmail.com (J.I.); erfanparand1380@gmail.com (E.P.); antonio.gasbarrini@unicatt.it (A.G.)

**Keywords:** inflammatory bowel disease, pathogenesis, immune system, gut microbiota, exposome, genetics, air pollution, water pollution

## Abstract

Inflammatory bowel diseases (IBDs), encompassing Crohn’s disease and ulcerative colitis, are complex chronic disorders characterized by an intricate interplay between genetic predisposition, immune dysregulation, gut microbiota alterations, and environmental exposures. This review aims to synthesize recent advances in IBD pathogenesis, exploring key mechanisms and potential avenues for prevention and personalized therapy. A comprehensive literature search was conducted across major bibliographic databases, selecting the most recent and impactful studies on IBD pathogenesis. The review integrates findings from multi-omics analyses, single-cell transcriptomics, and longitudinal cohort studies, focusing on immune regulation, gut microbiota dynamics, and environmental factors influencing disease onset and progression. Immune dysregulation, including macrophage polarization (M1 vs. M2) and Th17 activation, emerges as a cornerstone of IBD pathogenesis. Dysbiosis, as a result of reduced alpha and beta diversity and overgrowth of harmful taxa, is one of the main contributing factors in causing inflammation in IBD. Environmental factors, including air and water pollutants, maternal smoking, and antibiotic exposure during pregnancy and infancy, significantly modulate IBD risk through epigenetic and microbiota-mediated mechanisms. While recent advances have supported the development of new therapeutic strategies, deeply understanding the complex dynamics of IBD pathogenesis remains challenging. Future efforts should aim to reduce the burden of disease with precise, personalized treatments and lower the incidence of IBD through early-life prevention and targeted interventions addressing modifiable risk factors.

## 1. Introduction

Inflammatory bowel disease (IBD), encompassing Crohn’s disease (CD) and ulcerative colitis (UC), is a complex condition characterized by chronic inflammation of the gastrointestinal tract. The pathogenesis of IBD is multifactorial and still not completely clarified. Interactions among genetic predispositions, environmental influences, immune system dysfunctions, and alterations in the gut microbiota are involved. Gaining insight into such topics is of paramount importance for unraveling the mechanisms underlying IBD and developing effective therapeutic strategies and potentially for implementing effective prevention strategies [1].

The mainstay of IBD pathogenesis lies in a dysregulated immune response to gut microbiota. In healthy individuals, the immune system maintains a delicate balance that allows for tolerance to commensal microbes while mounting an appropriate response to pathogenic organisms. However, in genetically susceptible individuals, this balance is disrupted. Environmental factors such as diet, infections, and stress can exacerbate this dysregulation, leading to an inappropriate inflammatory response. This inflammatory milieu is primarily driven by T-helper (Th) cell responses. Th1 and Th17 cells are particularly implicated in Crohn’s disease and ulcerative colitis, respectively. The activation of these cells results in the production of pro-inflammatory cytokines that perpetuate tissue damage and inflammation [2].

Genetic factors also play a significant role in IBD susceptibility. Numerous susceptibility loci have been identified through genome-wide association studies, indicating that specific genetic variations can influence an individual’s immune response and microbiome composition. For instance, genes associated with autophagy and immune regulation are critical in maintaining intestinal homeostasis. However, genetic predisposition alone does not account for the entirety of disease variance; environmental triggers are essential in modulating these genetic risks [3].

The gut microbiota further complicates the pathogenesis of IBD. Dysbiosis, or an imbalance in microbial communities within the gut, has been consistently observed in IBD patients. This dysbiosis is characterized by reduced microbial diversity and an increase in pathogenic bacteria, which can breach the intestinal barrier and activate immune responses. The interplay between dysbiosis and immune dysfunction creates a feedback loop that exacerbates inflammation and contributes to the chronic nature of IBD [4].

Finally, the exposome—encompassing all environmental exposures—intersects with the other mentioned factors to influence disease outcomes. Factors such as dietary habits, antibiotic use, and exposure to pollutants can significantly impact gut health [5].

The pathogenesis of IBD is a multifaceted interplay between genetic susceptibility, environmental triggers, immune system dysregulation, and gut microbiota alterations. Recent advances in research have shed light on these aspects, revealing new avenues also for therapeutic interventions. This review discusses recent studies, emerging fields of research and novel insights that continue to enhance our understanding of IBD pathogenesis. Gaining insight into these topics is crucial for developing effective therapeutic strategies and exploring preventive approaches. Furthermore, a deeper understanding of pathogenetic mechanisms could pave the way for novel tools to identify patients with severe prognoses or therapy resistance at an early stage. Last but not least, novel biomarkers for non-invasive disease monitoring could emerge [6].

Building on these recent advances, this review delves deeper into the multifaceted pathogenesis of IBD, examining the interplay between the immune system, gut microbiota alterations, genetic predispositions, and environmental factors. Each section integrates established knowledge with the latest discoveries, offering novel perspectives that could inform therapeutic strategies, diagnostic tools, or preventive approaches.

## 2. The Innate and Adaptive Immune System

An aberrant immune response against epithelial elements and the intestinal microflora is the point of convergence of all the pathogenetic mechanisms of IBD [7].

The immune system includes two main compartments: innate and adaptive responses. The advent of single cell transcriptomics has revolutionized the investigation of the immune system, particularly T-cell populations. Recent studies indicate that, in the context of IBD, while the epithelial and stromal compartments undergo significant transcriptional shifts, the immune system exhibits profound alterations in both cell composition and abundance [8].

### 2.1. Innate Immune System

The epithelial layer, encompassing enterocytes and goblet cells, is the first gate of innate immunity. It plays a protective role thanks to its barrier function against pathogens [9]. Furthermore, the depletion or destruction of the mucus layer has been associated with the development of UC [10]. Previous studies have already highlighted that intestinal goblet cells play a role in the onset of Th17-based colitis, by delivering soluble antigens to CD103^+^ dendritic cells. Recent evidence shows that the mucus reduction observed in UC is associated with a reduction in both the number and size of goblet cells. Furthermore, reduced glycosylation of the mucin 2 (MUC2) protein amplifies the inflammatory response to *E. coli*, driven by NF-kB signaling [11]. Evidence from single-cell RNA sequencing suggests that mucosal barrier malfunctioning may stem from the production of ectopic mucin. Specifically, transcripts of mucin 1 (MUC1), a mucin typically produced in the stomach, have been identified in the colonic mucosa of patients with CD [8]. Remarkably, recent studies based on single-cell transcriptomics show that ileal epithelial cells from pediatric patients with treatment-naïve CD have an overexpression of S100A9, a calprotectin subunit. This finding underscores the direct involvement of the epithelial barrier to the immune process underlying IBD [12].

Among the cellular populations involved in innate immunity, innate lymphoid cells (ILCs) are coming to the spotlight. ILCs are circulant and resident lymphocytes with a role in regulating tissue homeostasis and immune responses [13]. Various subtypes of ILCs have been identified, including ILC1s, which are primarily expressed in the upper gastrointestinal tract, and ILC3s, predominantly found in the ileum and colon [14]. Within this cellular subset, the enigmatic and controversial role of ILC3s is being investigated. ILC3s produce IL-17 and IL-22 and express receptors for IL-1 and IL-23 [15]. On one side, an imbalance in the activity of ILC3s is associated with reduced gut barrier integrity and increased susceptibility to inflammation. For example, Bao and colleagues recently demonstrated that, in a dextran sodium sulfate (DSS) mouse model of colitis, the exposure to bacterial sphingolipids leads to a reduced IL-22 secretion from ILC3s with consequent worsening of colitis [16]. On the contrary, in other models of colitis (such as anti-CD40 antibody-induced acute colitis), the inflammation is driven by the IL-22 produced by ILC3s [17]. Furthermore, specific subsets of ILC3s, such as NKp44^+^, have a protective effect against CD and UC [18].

Macrophages, key players in innate immunity, mainly arise from monocytes and can be classified into M1 (inflammatory) and M2 (anti-inflammatory) types. M1 macrophages, activated by cytokines like IFN-γ and TNF-α, release cytokines (IL-12, IL-23) and reactive species that drive inflammation and pathogen clearance. In the gut, they disrupt tight junctions and induce epithelial cell apoptosis, worsening inflammation. M2 macrophages, marked by IL-4, IL-10, and CD206, promote tissue repair and resolve inflammation [19,20]. In UC, especially during the active phase, most macrophages in the lamina propria exhibit the M1 phenotype, which contributes to mucosal damage by disrupting tight junction proteins, compromising the epithelial barrier, and inducing apoptosis in epithelial cells. This results in heightened inflammation and further exacerbation of the disease [21]. On the contrary, increasing the proportion of M2 macrophages can alleviate symptoms of colitis in murine models, suggesting their protective role in IBD [22,23]. Novel insights on the role of macrophage polarization derive from single-cell and spatial transcriptomics. Garrido-Trigo and colleagues, upon identifying M0 macrophages within the colonic mucosa, highlighted significant variability in the transcriptional profiles of M1 macrophages in patients with IBD. Three major categories of M1 macrophages were identified: M1 ACOD1 (aconitate decarboxylase 1), M1 CXCL5 (CXC ligand 5), and the IDA (inflammation-dependent alternative) macrophage cluster. Notably, M1 macrophages in patients with UC (but not CD) exhibited elevated expression of neuregulin 1, a gene belonging to the epidermal growth factor family [24].

Myeloid cells, mainly neutrophils and macrophages, are also responsible for S100A9 calprotectin subunit. The role of calprotectin as an IBD biomarker has been known for some time [25]. However, recent evidence on the role of S100A9 in contributing to IBD pathogenesis is coming to the spotlight. In fact, S100A9 acts as an alarmin, a type of signaling molecule that is released during cellular stress or injury. It can activate immune responses by binding to Toll-like receptor 4 (TLR4) and other receptors, leading to the production of pro-inflammatory cytokines such as IL-1β, IL-6, and TNF-α. Furthermore, it also contributes to further neutrophils’ recruitment. Notably, S100A9 may also interact with the gut microbiota, promoting dysbiosis. These features suggest that S100A9 could represent a new actor in the pathogenesis of IBD [26,27].

**Figure 1:**
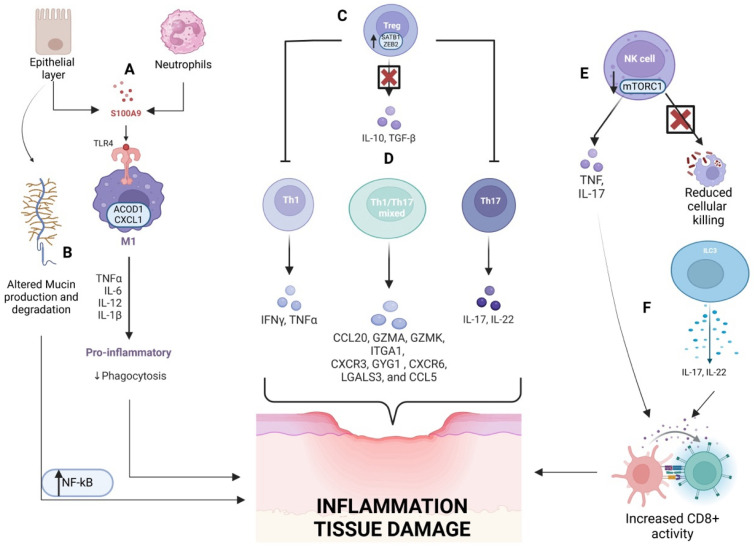
Dysregulated immune responses contributing to inflammation and tissue damage in IBD pathogenesis. Epithelial barrier disruption and neutrophil recruitment trigger the release of S100A9, which activates Toll-like receptor 4 (TLR4) signaling in M1 macrophages. These macrophages produce pro-inflammatory cytokines (TNFα, IL-6, IL-12, IL-1β), promoting inflammation and reducing phagocytosis (**A**). Altered production and glycosylation of mucin molecules contribute to amplify inflammation via NFkB (**B**). Dysregulated T-cell responses include reduced regulatory T cell (Treg) activity (**C**) and increased Th1, Th17, and mixed Th1/Th17 responses, producing IFNγ, TNFα, IL-17, and IL-22. These cytokines induce recruitment of inflammatory cells and expression of chemokines (CCL20, GZMA, ITGA1, etc.), amplifying tissue damage (**D**). NK cells with impaired mTORC1 signaling show reduced cytotoxicity, further aggravating inflammation (**E**). Innate lymphoid cells (ILC3) produce IL-17 and IL-22, enhancing CD8^+^ T cell activation and exacerbating tissue injury (**F**).

Natural killer (NK) cells, involved in both innate and adaptive immunity, recognize stress-induced and virus-infected cells. Found in the gut epithelium and stroma, they interact with various cell types and encounter antigens from microorganisms and the microbiota. In a healthy state, NK cells support immune responses by producing IFN-γ, enhancing anti-bacterial defenses [28]. In IBD patients, peripheral NK cells are dysregulated and produce high levels of pro-inflammatory cytokines, such as IL-17A and TNF-α, but exhibit diminished killing abilities. Additionally, these NK cells show reduced mitochondrial mass and impaired oxidative phosphorylation, indicating compromised cellular metabolism. The activity of mTORC1 (mammalian target of rapamycin complex 1), a key metabolic regulator, is also limited in both resting and cytokine-stimulated NK cells, contributing to their dysfunctional state in IBD. In CD, mucosal NK cells expressing NKp44 and NKp46 produce pro-inflammatory cytokines like IFNγ upon activation by intestinal macrophages, contributing to local inflammation. Conversely, in UC, NK cells appear to regulate the Th1/Th2 balance, with NKG2D^+^ NK cells playing a potential regulatory role in modulating Th2-mediated responses [29]. The mechanisms of the innate immune system implicated in IBD pathogenesis are summarized in Figure 1.

### 2.2. Adaptive Immune System

Unlike the innate immune response, which acts as the first line of defense, the adaptive immune system is characterized by its ability to recognize specific antigens and mount a tailored response. In IBD, this response often becomes dysregulated, leading to chronic inflammation. The pathogenesis of IBD is thought to involve an inappropriate immune reaction against the gut microbiota and dietary antigens in genetically susceptible individuals. Key players in the adaptive immune response include various T-cell subsets, such as T helper cells (Th1, Th2, Th17), cytolytic CD8^+^ cells, and regulatory T cells (Tregs). An imbalance between pro-inflammatory and anti-inflammatory Tregs is the mainstay of IBD pathogenesis [30,31].

Th1 polarization remains a mainstay of immune activation in IBD. Recent evidence obtained with single-cell transcriptomics confirms the presence of IFNG^+^ TNF^+^ T cells in mucosal and blood samples of patients with IBD, especially CD [32]. However, over the last years, increasing attention has been given to the role of Th17. In inflamed biopsies from patients with IBD, Medina and colleagues found a predominance of Th17 polarized lymphocytes overexpressing STAT3 (signal transducer and activator of transcription 3) and IL23R. Interestingly, according to single-cell transcriptional analysis, several lymphocytes showed a hybrid Th1/Th17 transcriptional profile, expressing genes such as CCL20 (C-C motif chemokine ligand 20), GZMA (granzyme A), GZMK (granzyme K), ITGA1 (integrin subunit alpha 1), CXCR3 (C-X-C motif chemokine receptor 3), GYG1 (glycogenin-1), CXCR6, LGALS3 (Galectin-3), and CCL5. Such cellular subpopulation was more abundant in inflamed tissues rather than non-inflamed areas and biopsies from healthy controls [33]. New transcriptional factors associated with the stimulation or inhibition of Th17 differentiation are being investigated. For example, the transcription factor ELF4 can both suppress inflammatory Th17 cell activity and induce macrophage M2 polarization [34].

It must be mentioned that a recent study by Tanemoto and colleagues proved the existence of a cellular subpopulation presenting mixed CD4/CD8 transcriptional activity. Such cells are closer to the CD4^+^ population in terms of transcriptional analysis but also express CD8-related transcripts such as perforins or granzymes [35]. Activated cytotoxic CD8^+^ T cells contribute to the initiation and progression of IBD. Several subtypes of CD8^+^ are known, including conventional cytotoxic (or cytolytic) T lymphocytes (CTLs) and CD8^+^ regulatory T cells [36]. Extensive insight into the role of CD8^+^ in UC is provided by Corridoni et al. thanks to single-cell transcriptomics. On one side, CD8^+^ lymphocytes are implicated in tissue damage through the production of TNF-α and other pro-inflammatory cytokines; on the other hand, some post-effector CD8^+^ T cells exhibit regulatory characteristics that may help mitigate excessive inflammation. Furthermore, the study also identifies terminally differentiated dysfunctional CD8^+^ T cells that express IL-26, a cytokine shown to attenuate acute colitis severity in a humanized mouse model [37]. CD8^+^ cells have been found to be related to disease activity, especially in CD. In fact, both in blood and intestinal samples of patients with active CD, CD39-expressing CD8^+^ T cells often showed signs of exhaustion, meaning they were less effective at responding to infections or inflammation [38].

Finally, an imbalance in the number and functionality of T regulatory lymphocytes is associated with the onset of IBD. Foxp3^+^ Treg cells are essential for suppressing inflammation, primarily through the production of IL-10 in response to TGFβ1 (transforming growth factor beta 1) stimulation [39]. Interestingly, it has been recently postulated that diets with high amounts of fatty acids can induce ferroptosis of such cellular subtype, leading to colitis worsening [40]. Surprisingly, single-cell transcriptional analysis in mucosal samples of patients with UC shows that Tregs are numerically increased in comparison to healthy controls. This could be related to the need for dampening inflammation. Conversely, transcriptional profiles of this cellular lineage are altered in UC, with the overexpression of transcripts (including SATB1, special AT-rich sequence-binding protein-1, and ZEB2, Zinc finger E-box-binding homeobox 2) that should be instead be suppressed for normal Treg functioning. It has to be remarked that, according to new evidence, tissue and circulating Tregs are not the only to be considered: in fact, mesenteric lymph nodes represent a critical site of effect of Tregs [41,42]. The key mechanisms of the adaptive immune system contributing to IBD pathogenesis are illustrated in Figure 1.

Given the critical role of the immune system in the pathogenesis of IBD, innovative therapeutic strategies are being explored to modulate immune responses and promote healing, one of which involves the application of mesenchymal stem cells (MSCs). MSCs possess unique properties, including immunomodulation, anti-inflammatory effects, and tissue regeneration, which make them suitable for addressing the complex pathogenesis of IBD. In Crohn’s disease, MSC therapy has received Food and Drug Administration approval for use in refractory cases, especially for complications such as fistulas and sphincter insufficiency. The mechanism of action of MSCs involves the secretion of bioactive factors that modulate the immune response, reduce inflammation, and promote healing of damaged intestinal tissue. They achieve this by downregulating pro-inflammatory cytokines and enhancing anti-inflammatory pathways, thereby restoring intestinal homeostasis. Moreover, MSCs exhibit low immunogenicity, allowing them to evade immune detection and reducing the risk of adverse reactions [43,44,45].

## 3. Gut Microbiota

Nowadays, the dissemination of integrated multi-omic analysis allows us to define disease-specific signatures. Common and recognizable profiles that characterize and distinguish IBDs are defined. For example, a recent study provides a multi-omic-based definition of gut microbiota, mycobiota, and metabolic signature in UC [46].

From an early age, the composition of microbiota is influenced and modified by several factors, including exposure to antibiotics. A systematic review has evaluated the impact of antibiotics on the infant gut microbiome composition and resistome in infants in low- to middle-income countries settings. Antibiotics disrupt microbiome diversity and taxonomy, with effects likely mediated by antibiotic class, duration of administration, and follow-up time [47]. The association between antibiotic exposure and an increased risk of developing IBD has been confirmed by a recent meta-analysis involving 99,104 IBD patients and 2,273,336 controls, reporting an odds ratio (OR) of 1.66 (95% CI, 1.28–2.16) [48]. Notably, exposure to cephalosporins was strongly associated with new-onset IBD (OR, 1.62; 95% CI, 1.26–2.08), as was exposure to penicillins (OR, 1.50; 95% CI, 1.14–1.98) and macrolides (OR, 1.50; 95% CI, 1.14–1.98). Similarly, quinolones (OR, 1.49; 95% CI, 1.45–1.54), tetracyclines (OR, 1.49; 95% CI, 1.44–1.54), and metronidazole/tinidazole (OR, 1.34; 95% CI, 1.30–1.38) were significantly linked to an increased IBD risk [47]. In children, the cumulative frequency of antibiotic use plays a critical role in the risk of developing IBD. While a single dispensation within the year preceding diagnosis was not significantly associated with IBD risk, cumulative exposure—defined as two or more dispensations within the same timeframe—was linked to a significantly higher risk. Prolonged antibiotic exposure further increases the risk of developing IBD, with an odds ratio (OR) of 1.49 (95% CI, 1.12–1.98) for exposure over three years and 1.46 (95% CI, 1.37–1.55) for exposure over two years [48]. Data from Sweden and Norway further underscore the importance of early antibiotic use. Among 103,046 children, 395 developed IBD, with a higher risk observed in those exposed to antibiotics during the first year of life (HR = 1.33; 95% CI, 1.01–1.76) compared to those without exposure. In contrast, the frequency of infections did not show a significant association with IBD risk [49].

The composition of microbiota in IBD patients significantly differs compared to healthy individuals. Beta-diversity, defined as the difference in types of microbes between multiple samples, and alpha-diversity, defined as the difference within an ecosystem or a sample, are both reduced in microbiota of IBD patients compared to healthy individuals, particularly if looking at the stability of the microbiome of patients over time [50]. As confirmed by recent meta-analysis, which has included 13 studies, reduced alpha-diversity is a consistent feature of both CD and UC but was more pronounced in CD (OR of 3.20, 95% CI: 2.09–4.88, *p* < 0.001) [51]. More in depth, key alterations include a decrease in beneficial *Firmicutes* and *Bacteroidetes*, alongside an increase in pathogenic *Proteobacteria*. Recent studies have confirmed such trend reporting that, specifically, the abundance of *Faecalibacterium prausnitzii* and *Roseburia intestinalis*, both known for their anti-inflammatory properties, is markedly reduced in IBD patients, while *Escherichia coli* and other members of the *Proteobacteria* phylum show increased prevalence, correlating with disease severity and inflammation levels [52,53].

**Figure 2:**
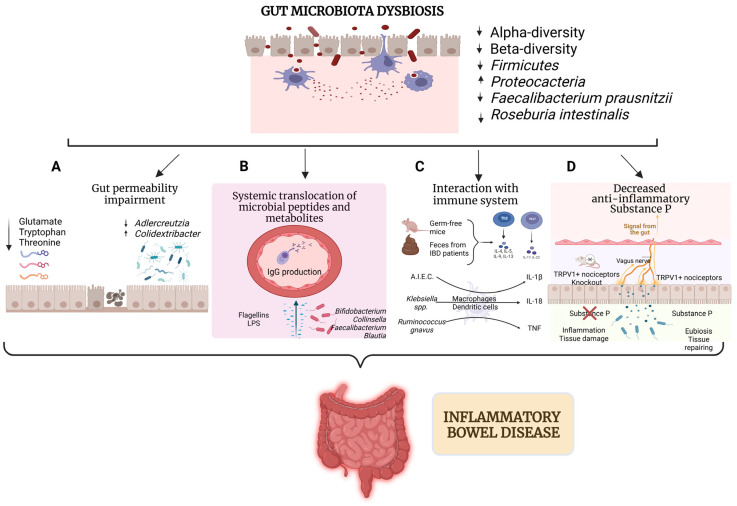
The impact of gut microbiota dysregulation on IBD pathogenesis. Reduced microbial diversity, depletion of beneficial taxa such as *Faecalibacterium prausnitzii* and *Roseburia intestinalis*, and enrichment of pathogenic taxa like *Proteobacteria* are the core features of gut dysbioisis. This disruption impairs gut permeability by reducing key amino acids and altering microbial composition, allowing microbial components to penetrate the intestinal barrier (**A**). Translocated microbial peptides and metabolites, such as flagellins and LPS, enter systemic circulation, triggering IgG production and amplifying inflammation (**B**). Immune system activation involves increased inflammatory cytokine production following exposure to microbial products, as observed in germ-free mice receiving fecal transplants from IBD patients. Pathogenic bacteria, including *Klebsiella* spp. and *Ruminococcus gnavus*, further stimulate macrophages and dendritic cells to release pro-inflammatory cytokines like IL-1β, IL-18, and TNF (**C**). Additionally, dysbiosis disrupts TRPV1^+^ nociceptor signaling, reducing levels of the anti-inflammatory mediator Substance P. This impairment compromises vagus nerve-mediated tissue repair, enhances inflammation, and deranges pain perception (**D**).

Gut microbiota contributes to determining intestinal inflammation mainly through its interaction with the immune system (Figure 2). For example, germ-free mice colonized by gut microbiota from IBD patients show an elevation of Th17 and Th2 cells, with concomitant reduction of RORγt^+^ Treg cells [54]. Interestingly, recent evidence shows that adherent-invasive *Escherichia coli* (AIEC) has the ability to attach to and penetrate epithelial cells, stimulating the secretion of interleukin-1 beta (IL-1β) from macrophages, which stimulates the differentiation of Th17 cells; in the same way, *Klebsiella pneumoniae* and *Klebsiella aerogenes* enhance the release of pro-inflammatory cytokines (particularly IL-1β and IL-18) from dendritic cells and macrophages, promoting the differentiation of Th1 and Th17 cells. Moreover, mucolytic bacteria like *Ruminococcus gnavus* are able to produce polysaccharides that stimulate tumor necrosis factor (TNF) secretion from dendritic cells [55]. Similarly, mice administered with DSS show more elevated IL-6 and IL-1β serum concentrations and monocyte-chemoattractant protein (MCP)-1 when receiving oral gavage of *C. difficile* after the DSS [56].

As mentioned, the intestinal mucus layer plays a central role in maintaining gut homeostasis. Understanding how gut microbiota interacts with mucus layer could represent a key factor in elucidating IBD pathogenesis. To such a purpose, a recent study demonstrated that mice deficient in the MUC2 protein also exhibit specific microbiota features impacting colitis severity [57]. Furthermore, the administration of *Lactobacillus reuteri* leads to a mitigation of colitis symptoms in DSS-induced colitis mice. This effect is mediated by the synthesis of indole-3-acetic acid (IAA), a tryptophan derivate, and to the thickening of the colonic mucus layer [58]. The mucus layer is not the only constituent of intestinal permeability. The association between gut microbiome composition and impaired barrier function is a mainstay of the IBD pathogenesis. Recent evidence suggests that decreased prevalence of *Adlercreutzia* (with anti-inflammatory properties), coupled with increased abundance of *Colidextribacter* (with a role in cellular oxidative stress), are related to impaired intestinal permeability. Moreover, the depletion of microbial pathways associated with the biosynthesis of the amino acids glutamate, tryptophan, and threonine suggests the role of microbiota-metabolites in driving altered gut permeability [59].

Gut microbiota could induce damage even outside of the gut. In fact, translocation of pathogens from mucosal surfaces into peripheral tissues can directly stimulate immune cells to produce many proinflammatory cytokines, increasing intestinal inflammation [60] (Figure 2). A cross-sectional study focused on characterizing the adaptive immunoglobulin response to human-derived microbiota flagellins in CD and UC. In particular, patients with CD, but not patients with UC, show increased serum IgG antibody responses to human ileal-localized *Lachnospiraceae flagellins*, with a subset of patients that also show increased CD4 cells against such antigens [61]. More recently, a correlation between a subset of commensal gut microbiota constituents that translocate across the gut barrier and systemic immunoglobulin G (IgG) responses has been described in mice and humans. In particular, the systemic IgG ranges were detectable against translocating gut bacteria and taxa that can be proinflammatory, such as *Bifidobacterium*, *Collinsella*, *Faecalibacterium*, and *Blautia* genera. In addition, patients with CD exhibited greater IgG targeting of gut commensals than UC patients [62].

The taxonomical impairment in gut microbiota results in a shift in the metabolomic profile of IBD patients. Functional modifications also play an important role in developing IBD [63]. The most relevant modifications are represented by higher levels of organic acids and amino acids such as tryptophan, glutamine, arginine, 5-hydroxytryptophan, and histidine, along with a decrease in short-chain fatty acids (SCFA) [64]. However, the role of these metabolites in IBD is still unsolved. The latest evidence reveals that a mixture of SCFA-producing bacterial strains attenuates UC in DSS-mice models by inducing M2 macrophage polarization and inhibiting JAK/STAT3/FOXO3 axis activation [65].

In recent years, focus has been given to the so-called “neglected microbiome” (fungi, protozoa, and viruses), which have also shown a great relevance in the IBD pathogenesis. Regarding the gut virome, *Siphoviridae* and *Myoviridae* (which belong to the order of *Caudovirales*) are significantly enriched in IBD patients, while *Quimbyviridae* are decreased [66]. Specific changes have also been highlighted between CD and UC patients in terms of virome. Patients with CD exhibit greater prevalence of the *Hepeviridae* family and a reduction of the *Virgaviridae* family; conversely, UC patients show an abundance of transcripts encoded by *Orthoepadnaviridae* [67]. The alterations in the virome significantly contribute to the pathogenesis of IBD through different pathways, such as altering the integrity of the intestinal mucosa; stimulating the immune response by influencing the host bacteria; releasing bacterial components after lysis or transcytosis; and inducing IFN type-I response in immune cells, which can worsen intestinal inflammation and increase the severity of the disease [68]. A recent study demonstrates how bacteriophage can influence the bacteria microbiota. Monocolonized germ-free mice with *Bacteroides fragilis* NCTC 9343 in the presence of Barc2635, a lytic double-stranded DNA bacteriophage belonging to the *Caudoviricete*, show a switch of the polysaccharide A (PSA) promoter of *B. fragilis* in the “OFF” state causing reduced a decrease in Tregs with a consequent reduction in inflammation [69]. Fecal virome transplantation (FVT) experiments using human fecal virus-like particles (VLPs) in a mouse model confirmed the roles of IBD viral signatures in disease progression. The expression levels of E-cadherin, β-catenin, and occludins were upregulated in the HF-FVT (Healthy Donor Fecal Virome Transplantation) group and downregulated in the IBD-FVT group, indicating worsened gut permeability in the IBD-FVT group. Additionally, the IBD-FVT group exhibited significantly greater levels of inflammatory cytokines, including TNF-α, IL-6, IFN-γ, and IL-1β. These findings indicate that the colonization of viruses from healthy donors has a mitigating effect on colitis in a mouse model, whereas that of viruses from IBD patients exacerbates colitis with a substantial expansion of IBD-enriched viral operational taxonomic units (vOTUs) within the mouse gut microbiota [66]. The rise of high-throughput sequencing also allowed a better understanding about fungal dysbiosis and the implication in the pathogenesis of IBD. *Didymellaceae*, *Saccharomycetales*, *Malassezia*, *Wickerhamomyces*, *Cutaneotrichosporon*, *Saccharomyces*, *Clavispora*, *Alternaria*, and *Candida* are major fungal markers in UC [46]. In 2,4,6-trinitrobenzenesulfonic acid (TNBS)-derived colitis mice models, fungi depletion protects against the progression of the disease. The mechanisms through which fungi promote pro-inflammatory response in IBD relies on CD4^+^ T cells, which are stimulated to produce IFN-γ, IL-17A, and TNF-α [70]. The loss of integrity of IECs through the disruption of tight junction (TJ) Occludin and ZO-1 lead to the penetration of pathogens like fungi and bacteria into the mucosal barrier and consequently activate TLRs, Dectin-1, and caspase-recruitment domain 9 (CARD9), inducing a more severe inflammatory phenotype [71].

Finally, gut microbiota could play a role in determining IBD susceptibility, even through the interaction with the peripheral nervous system (PNS) (Figure 2). For example, a recent study analyzed the role of TRPV1^+^ nociceptors in regulating the composition of the intestinal microbiota through substance P, which limits inflammation and promotes intestinal tissue protection, restoring intestinal homeostasis. In particular, in the absence of TRPV1^+^ nociceptors, vancomycin-sensitive Gram-positive bacterial communities lead to enhanced susceptibility to DSS-induced colitis in mice. Moreover, mono-colonization of a consortium of Gram-positive bacteria *Clostridium* spp. in germ-free mice with disrupted nociception was able to exacerbate intestinal inflammation. In contrast, mono-colonization of nociceptor-sufficient germ-free mice with *Clostridium* spp. was tissue protective. Mechanistically, disruption of nociception resulted in decreased levels of substance P, and the therapeutic delivery of substance P promoted tissue-protective effects exerted by TRPV1^+^ nociceptors in a microbiota-dependent manner [72].

Building on the growing understanding of the gut microbiota’s role in inflammation and disease, fecal microbiota transplantation (FMT) has gained attention as a therapeutic strategy to restore microbial balance by transferring fecal microbiota from healthy donors to recipients. FMT has demonstrated its effectiveness in *Clostridioides difficile* infection [73]. Starting from this, FMT has been tested as a new promising therapeutic strategy in IBD. As evidence of the role of the gut microbiota as a trigger of inflammation, studies have been performed on FMT in patients with mild-to-moderate UC, demonstrating effectiveness in inducing clinical remission, despite the heterogeneity in design protocols, regarding routes of FMT administration, inclusion criteria, and follow-up periods. These results confirmed the possible use of FMT as a therapeutic option in UC patients. Nevertheless, the insufficient data about long-term effects and the variability in study designs currently do not allow FMT to be recommended as a standard treatment in clinical practice [73,74]. The efficacy of FMT in CD remains unclear. Some studies suggest a potential role in the induction and maintenance of clinical response and remission. However, the lack of randomized clinical trials and robust data currently does not support the application of FMT in CD patients [75].

## 4. Genetics

Genetics has long been recognized as a predisposing factor in the development of IBD, but its role has evolved over the years. While the focus on genetic factors may not be as prominent as it once was, recent studies continue to reveal intriguing insights into how genetic variations contribute to IBD susceptibility.

### 4.1. Polygenic Contributions to IBD

Several genes have been historically linked to an increased risk of IBD. Nucleotide Binding Oligomerization Domain Containing 2 (NOD2) was the first gene identified as a susceptibility factor for CD. It is involved in recognizing bacterial components and initiating immune responses. Variants in this gene impair autophagy, affecting bacterial clearance and immune regulation [76]. Among the genes associated with IBD, IL23R is notable for its protective alleles. The R381Q polymorphism reduces the risk of Crohn’s disease by approximately threefold, and additional rare protective variants, such as G149R and V362I, further support the gene’s protective role. These findings underscore the importance of IL23R in the IL23/Th17 signaling pathway, which plays a critical role in regulating CD4^+^ Th17 cell differentiation. These insights underscore the therapeutic potential of targeting this pathway in IBD. Mutations in autophagy-related genes, such as ATG16L1 (Autophagy-Related 16 Like 1) and IRGM (Immunity-Related GTPase M), have been linked to immune response dysregulation. Additionally, genes involved in interleukin signaling pathways, including STAT3, TYK2, and JAK2, as well as PTPN2 (Tyrosine-protein phosphatase non-receptor type 2), which is associated with other autoimmune diseases, have been shown to contribute to the development of IBD [77].

An interesting genetic locus possibly involved in the predisposition to IBD is OCTN1/Slc22a4. OCTN1 (organic cation transporter, novel, type 1) is an organic cation transporter expressed in various tissues throughout the body. It is particularly abundant in intestinal epithelial cells, especially in the small intestine and colon, where it plays a crucial role in the absorption of specific molecules. Additionally, OCTN1 was also found in the proximal tubules of the kidney, liver, skeletal muscle, and placenta. In the gut, OCTN1 could be involved in the traffic of bacterial molecules, contributing to IBD pathogenesis. Polymorphisms in OCTN1 had been previously associated with predisposition of IBD (UC in particular) and occurrence of GI cancer in young individuals. Furthermore, OCTN1 facilitates the absorption of ergothioneine, a key antioxidant that protects intestinal cells from oxidative stress. When OCTN1 is dysfunctional, antioxidant protection is reduced, making the gut more vulnerable to inflammation. Recent evidence has shed new light on the role of this genetic locus. According to Del Chierico and colleagues, OCTN1 deficient mice (OCTN1^−/−^) show reduced DSS-induced colitis severity and a higher baseline percentage of Treg, T memory, Th2, and Th17 cells [78,79,80,81].

CARD9 has recently emerged as a potential predisposing factor for IBD. This enigmatic protein interacts with Toll-like receptors (TLRs) to activate innate immunity via mitogen-activated protein kinases (MAPK) and nuclear factor kappa-light-chain-enhancer of activated B cells (NF-kB). The majority of CARD9 variants (such as rs10870077, rs4077515, and rs10781499) have a proinflammatory effect and represent predisposing variants to IBD. In contrast, uncommon variants such as rs141992399 and rs200735402 have an anti-inflammatory effect and act as protective from IBD [82]. It has been recently demonstrated that CARD9 also drives cellular apoptosis. In particular, CARD9-deficient mice show increased neutrophil apoptosis and are more susceptible to intestinal inflammation [83]. Finally, CARD9 is involved in the recovery from colitis through the production of IL-22 and other antimicrobial peptides [84]. Several other functions have been proposed for CARD9, including other immunomodulatory activities and control of tumorigenesis [82].

Polymorphisms and genetic variants of genes encoding for inflammatory cytokines have acquired recent relevance in the field of IBD susceptibility. TNF superfamily member 15 (TNFSF15) is the gene that encodes tumor necrosis factor-like cytokine 1A (TLA1), a member of the TNF cytokines superfamily. It interacts with its receptor DR3, encoded by the TNFRSF25 gene. Several roles are being investigated for TNFSF15 variants, including IBD susceptibility and fibrosis development [85]. More in depth, the binding of TNFSF 15 to DR3 results in increased bacterial uptake via PDK1 by phagocytic cells. Furthermore, the TNFSF 15-DR3 interaction leads to increased production of reactive oxygen species and reactive nitrogen species, as well as the activation of autophagy pathways. Taken all together, these modifications lead to increased intracellular bacterial clearance [86]. A Genome-Wide Association Study (GWAS) and a Transcriptome-Wide Association Study (TWAS), conducted on a Japanese population, identified a significant association between TNFSF15 expression in whole blood and the development of CD [87]. This association has been confirmed by a meta-analysis including eight studies and a total of 2682 patients. rs6478109 polymorphisms were significantly associated with the onset of CD (not UC), independent from disease phenotype [88].

### 4.2. Monogenic IBD

Monogenic forms of IBD, although rare, are typically severe and resistant to conventional therapies [89]. Unlike polygenic or idiopathic IBD, monogenic forms follow Mendelian inheritance patterns and are associated with specific genetic mutations [90]. Advances in next-generation sequencing have led to the identification of over 80 causal genes, IL10RA/IL10RB (interleukin 10 receptor alpha/beta), XIAP (X-linked inhibitor of apoptosis protein), CYBB (cytochrome b-245 beta chain), LRBA (lipopolysaccharide-responsive and beige-like anchor protein), and TTC7A (tetratricopeptide repeat domain 7A), which provide valuable insights into distinct disease mechanisms [91]. These mutations often disrupt key immune processes, underscoring the importance of precise genetic diagnosis. Mutations in genes regulating phagocyte functions—such as bacterial killing, cell migration, or inflammatory pathways—can drive monogenic IBD. For instance, antimicrobial activity is notably impaired in chronic granulomatous disease (CGD), which is linked to NADPH oxidase complex mutations. Nearly half of CGD patients develop intestinal inflammation, illustrating how genetic disruptions in microbial clearance can directly contribute to IBD pathogenesis [92]. Furthermore, recent discoveries, such as mutations in CYBC1 and Protein Kinase C Delta (PRKCD), have highlighted additional failures in microbial killing mechanisms that exacerbate disease severity [93,94]. Endothelial cell dysfunction also plays a critical role in some forms of monogenic IBD. Mutations in CD55, for example, enhance complement activation, which damages cells and amplifies inflammation [95]. Similarly, mutations in solute carrier organic anion transporter family member 2A1 (SLCO2A1) impair prostaglandin clearance, leading to chronic intestinal bleeding and further complicating disease management [93]. Together, these genetic abnormalities demonstrate the complex cellular disruptions underlying monogenic IBD.

Monogenic IBD is often characterized by early onset, atypical family history, unusual endoscopic findings, and extra-intestinal manifestations. These clinical features can help distinguish it from polygenic forms, enabling earlier and more targeted interventions. While emerging therapies such as hematopoietic stem cell transplantation and gene therapy using lentiviral vectors have shown promise, significant challenges remain in translating these advances into routine clinical practice [96]. Concerns about long-term safety and scalability highlight the need for continued research and validation of these novel approaches.

### 4.3. Genetics–Microbiota Interactions

Recent research has brought increasing attention to the dynamic interplay between host genetics and the gut microbiota in the pathogenesis of IBD. This relationship is bidirectional, with host genetic variations influencing microbial communities, while microbial proteins, in turn, modulate host gene expression. Understanding this complex interaction is key to uncovering novel pathways in IBD development and progression. Host genetic variations play a significant role in shaping the composition and functionality of the gut microbiota. For example, variants in genes such as IL17REL (interleukin 17 receptor E-like), MYRF (myelin regulatory factor), SEC16A (SEC16 homolog A, endoplasmic reticulum export factor), and WDR78 (WD repeat domain 78) have been linked to changes in microbiota diversity. In particular, MYRF variations are associated with reduced production of short-chain fatty acids (SCFAs), which are essential for maintaining gut health and mitigating inflammation [79]. These findings suggest that genetic predispositions can directly impact the microbial environment, creating conditions conducive to IBD. Conversely, microbial proteins can influence host gene expression, further complicating the immune response. For example, a study by Sudhakar and colleagues (2022) demonstrated how specific microbial proteins interact with host genes to modulate immune responses and inflammatory pathways in Crohn’s disease [97]. These interactions underscore the intricate feedback loop between the microbiome and host genetics, wherein dysbiosis not only results from but also exacerbates genetic vulnerabilities.

This evolving understanding of genetics–microbiota interactions has significant implications for IBD treatment. It highlights the potential for integrated therapeutic approaches that target both genetic and microbial contributors to disease. By combining genetic profiling with microbiota analysis, clinicians may be able to develop personalized treatment strategies that address the root causes of IBD.

## 5. The Exposome

Several modifiable environmental factors, collectively referred to as the “exposome”, have been investigated for their association with IBD risk [98]. While smoking remains the only well-established risk factor [99], evidence for other modifiable factors influencing disease susceptibility is steadily emerging, though preventive strategies have yet to be fully developed. We have comprehensively gathered all available evidence on environmental modifiable risk factors for CD and UC, categorizing them by life period—childhood or adulthood—in which they exert influence.

### 5.1. Childhood Exposures

Several prenatal and birth-related factors have been suggested to influence the development of CD or UC in various studies. Growing evidence suggests that in utero environmental exposures can influence epigenetic programming in the fetus, potentially shaping lifelong health risks, including susceptibility to inflammatory conditions [100,101,102]. These epigenetic modifications may also be inherited by future generations, representing transgenerational epigenetic inheritance [103,104]. For instance, studies have identified that an excessive intake of certain micronutrients, such as folate, vitamin B12, or methionine, in the maternal diet could increase the risk of colitis in offspring [105]. Additionally, maternal infections during pregnancy might trigger IL-6 production, leading to epigenetic changes in fetal intestinal epithelial stem cells and predisposing the child to inflammatory diseases [106]. These early-life exposures are part of the broader childhood exposome, as highlighted in a 2020 study by van der Sloot et al., which investigated 36 factors and identified prenatal smoking as a significant risk factor for CD (OR 1.89; 95% CI 1.38–2.59) and a nominally significant risk factor for UC (OR 1.61; 95% CI 1.16–2.23), even after accounting for smoking in later life [107]. Mechanistically, maternal smoking has been associated with persistent alterations in DNA methylation, miRNA dysregulation, and gut microbiome alterations in the exposed offspring [108,109,110].

In contrast, breastfeeding has been shown to exert a protective effect against CD (OR 0.71; 95% CI 0.59–0.85), UC (OR 0.78; 95% CI 0.67–0.91), and overall IBD (OR 0.74; 95% CI 0.66–0.83), with longer breastfeeding durations linked to lower disease risk [111,112]. Notably, this protective effect is stronger in Asian populations for CD (OR 0.31; 95% CI 0.20–0.48) compared to Caucasians, and in studies conducted before 2000, where IBD risk was reduced more significantly (OR 0.58 versus 0.82) [111]. Similarly, van der Sloot et al. identified a modest protective effect of breastfeeding on CD (OR 0.56; 95% CI 0.37–0.87) [107], while Gordon et al. (2022) reported that any form of breastfeeding, including mixed feeding, reduced UC onset risk (OR 0.48; 95% CI 0.25–0.93, *p* = 0.03), although no significant effect was observed for CD in twin pairs with IBD [113]. Breastfeeding’s protective effect is hypothesized to stem from its role in establishing a healthy gut microbiota and supporting innate mucosal immunity development [114,115]. Conversely, the absence of breastfeeding has been linked to increased *Clostridium difficile* colonization and other immune-mediated diseases, including IBD.

Similarly, other early-life factors, such as mode of delivery, have been investigated for their potential influence on IBD risk. The impact of cesarean delivery, however, has shown inconsistent findings, with some studies linking cesarean births to an increased risk of CD (OR 1.38; 95% CI 1.12–1.70), but not UC or overall IBD [116,117,118]. Other research has found no significant differences in the incidence of CD or UC between cesarean and vaginal deliveries [112]. In the same vein, factors such as preterm birth, birthweight, and birth length have not been consistently associated with IBD risk [107].

On the other hand, as previously discussed in the gut microbiota section, the use of antibiotics during pregnancy or early life has been implicated as a potential risk factor for IBD. Antibiotics may disrupt the gut microbiota, reducing its diversity and taxonomic richness while altering its metabolic function [119,120,121]. For example, antibiotics have been associated with an increased risk of CD (OR 1.74; 95% CI 1.35–2.23) and IBD overall (OR 1.57; 95% CI 1.27–1.94), with a dose–response effect observed in pediatric CD (OR 2.75; 95% CI 1.72–4.38) [122]. This impact is especially pronounced in CD, where antibiotics exacerbate dysbiosis, a characteristic feature of the disease [123,124].

The “hygiene hypothesis” has also been proposed to explain IBD pathogenesis, suggesting that reduced exposure to environmental microorganisms disrupts immune system balance, increasing susceptibility to autoimmune conditions. A meta-analysis found that living near farm animals (OR 0.46; 95% CI 0.20–0.72), having pets (OR 0.77; 95% CI 0.59–0.94), sharing a home (OR 0.49; 95% CI 0.25–0.75), bed-sharing (OR 0.54; 95% CI 0.43–0.65), and having two or more siblings (OR 0.93; 95% CI 0.88–0.98) were protective against CD [125]. Similarly, these factors, except having siblings, along with access to hot water (OR 0.76; 95% CI 0.57–0.95) and a personal toilet (OR 0.73; 95% CI 0.59–0.88), conferred protection against UC [126]. Conversely, urban living has been associated with a higher incidence of CD (IRR 1.42; 95% CI 1.26–1.60) and IBD overall (OR 1.35; 95% CI 1.15–1.58) [127]. However, more recent studies have not confirmed the association between sibling presence and IBD risk, although other hygiene-related markers still point to a protective pattern. Notably, pet ownership during early life was shown to exert a protective effect, particularly in the first year of life, against CD (OR 0.30; 95% CI 0.22 –0.40) and UC (OR 0.32; 95% CI 0.24–0.44), with significant effects persisting throughout childhood [107].

Infection with Helicobacter pylori appears to offer protection against IBD, with a stronger effect observed in children compared to adults (OR 0.24 vs. 0.46) [128,129,130,131]. This association is thought to be mediated by immune modulation, including Toll-like receptor 2 activation, interleukin-10 production, inhibition of type I interferon and interleukin-12 production, and regulatory T-cell accumulation [132,133,134]. Reduced exposure to infectious agents during childhood may limit microbiota diversity and promote a pro-inflammatory Th2 immune response, increasing IBD risk [119,135]. Additionally, maternal exposure to farm environments has been shown to increase both the number and function of cord-blood regulatory T cells [136].

Other factors associated with increased IBD risk include low fruit intake, low physical activity, appendectomy, frequent childhood gastroenteritis admission, previous exposure to antibiotics and certain vaccinations, and early dietary introduction of gluten and sugar [137,138]. Notably, Piovani et al., among seven vaccinations, found that only the poliomyelitis vaccine was associated with IBD in a small meta-analysis [139]. Similarly, Ungaro et al. reported a significant association between antibiotic exposure and an elevated risk CD (OR 1.74; 95% CI 1.35–2.23) and IBD overall (OR 1.57; 95% CI 1.27–1.94), but not UC. A dose–response relationship was identified, with a stronger effect observed in pediatric CD (OR 2.75; 95% CI 1.72–4.38). All antibiotic classes, except for narrow-spectrum penicillins, were linked to an increased risk of IBD [122]. The biological plausibility of these findings is supported by the known impact of antibiotics on the gut microbiota. Antibiotics reduce both the taxonomic richness and diversity of the microbiome [119,120], impairing its metabolic function [121]. This disruption appears more pronounced in CD compared to UC, as antibiotics have been shown to exacerbate the dysbiosis characteristic of CD [123,124]. Stressful life events, such as parental divorce or a family death, have also been implicated [140,141]. Chronic stress is a well-established risk factor for both IBD onset and exacerbation. Recent findings highlight the role of enterochromaffin cells (EC) in gut sensing mechanisms that influence anxiety-like behaviors, as well as the impact of chronic stress and elevated corticosteroid levels on inflammatory enteric glial cells, which in turn activate immune cells [142,143]. This cascade can lead to various physiological alterations in the intestine, including increased gastrointestinal secretion, increased intestinal permeability, reduced mucosal blood flow, and alterations of the gut microbiota composition, thereby increasing the risk of IBD [144,145,146]

### 5.2. Adulthood Exposures

Besides childhood, numerous environmental factors have been studied for IBD risk also in adulthood. One of the most common problems of the modern era is air pollution, and in recent years the interest in this topic has also grown in the field of IBD [144,147,148]. From a pathogenetic point of view, environmental pollutants can alter the redox balance at the level of the intestinal barrier, oxidizing the lipids of the intestinal epithelium [149,150]. Oxidative stress, moreover, can cause damage to the mucous layer of the gastrointestinal tract, leading to bacterial invasion, triggering immune responses, and initiating IBD [149]. It has been shown that exposure to PM2.5 increases the concentrations of oxidized low-density lipoproteins [151]. Furthermore, exposure to particulate matter and ozone modifies intestinal permeability by damaging the intercellular barriers [152]. Kaplan et al. showed that residential exposures to sulfur dioxide (SO_2_) and nitric oxide (NO_2_) might increase the risk of early-onset UC and CD, respectively [148]. Such exposures also seem to influence the course of the disease; an increase in the density of pollutant emission by 1-log was associated with a 40% increase in the rate of IBD hospitalizations (incidence RR = 1.40; 95% CI: 1.31–1.50, for both UC and CD hospitalization) [153]. Finally, in a large Italian study, long-term exposure to PM2.5 levels above 20 μg/m^3^ was associated with a significant increase in the development of IBD (adjusted OR of 1.21 95% CI: 1.03–1.42) compared to those below 20 μg/m^3^ [154]. Liu et al. analyzed (455,210 people aged 40–69 years) the long-term associations between exposure to air pollution and the risk of IBD [155]. They found significant associations for UC with PM2.5, PM2.5–10, PM10, NO_2_, and Nox. In contrast, Okafor et al. found no associations between PM2.5, O_3_, diesel PM, traffic density, and IBD [156].

Besides air, the ingestion of pollutants via water sources has also been recently investigated as a relevant factor for the incidence and course of IBD [157,158]. The presence of antagonists of steroid receptors, endocrine-disrupting chemicals (EDCs), and the interaction with the glucocorticoid receptor and peroxisome-proliferator-activated receptor gamma (PPAR-γ) may play an important role in the pathogenesis of IBD through in vitro studies. However, there are no supporting in vivo studies. Concerning metal elements and disinfectants in drinking water, they have been associated with alterations in the microbial environment in the intestinal tract and exacerbate immune system responses [159]. Some observational studies have shown that iron nitrate and sulfite in drinking water is associated with a higher incidence of IBD [160,161,162]. A recent large Chinese study found an association between increased concentrations of manganese, mercury, selenium, sulfur tetraoxide (SO_4_), chlorine, and nitrate nitrogen (NO_3_-N) and a higher risk of IBD [163].

Among environmental risk factors, cigarette smoking stands out as one of the most well-established, with distinct effects on UC and CD [99,164]. In UC, smoking seems to modulate the immune system and reduce the production of TNF-α through the action of nicotine on the nicotinic acetylcholine receptor. However, the mechanism by which it exerts its protective effect in UC has not been clearly defined [164]. In CD, smoking can increase carbon monoxide (CO), causing an alteration of the vasodilation capacity in microvessels, resulting in ischemia and perpetuation of ulceration and fibrosis [165]. In CD, smoking has been associated with both the development of the disease and its course, particularly for CD. A prospective study including 229,111 women, showed that smoking increases the risk of developing CD among current smokers with a HR of 1.90 (95% CI: 1.42–2.53) [166]. The increased risk is associated with the number of packs smoked. It also seems to increase the risk of penetrating intestinal complications, as well as the need for surgery [99,167]. Notably, smoking cessation has been associated with a reduction in disease risk, as former smokers show a lower HR of 1.35 (95% CI: 1.05–1.73) compared to active smokers (HR 1.90, 95% CI: 1.42–2.53) [166]. Additionally, smoking cessation positively impacts disease progression, leading to improvements such as a reduction in disease flares, decreased reliance on steroids, and less frequent need for immunosuppressive therapy [168,169].

In this context of pathogenic environmental factors in IBD, sleep disorders have been investigated and are emerging as potential risk factors (OR: 3.09) for IBD development, exacerbation, and diminished quality of life [170]. Sleep disorders were observed in 59.6% of patients with IBD, which was significantly higher than in healthy individuals [171]. It has been hypothesized that they may not only be a result but rather a cause of chronic inflammatory diseases. In support of this hypothesis, alterations in some non-rapid eye movement (NREM) sleep stages, where the greatest impact of immune regulation and the decrease in colonic contractility occurs, can lead to a reduction in mucosal integrity [172]. Furthermore, in healthy young volunteers with disturbed sleep, increases in key cytokines involved in the pathological processes of IBDs, such as interleukins IL-1β, TNF-α, and IL-6, have been found [171]. However, there is a scarcity of longitudinal studies that investigate sleep disorders in the pathogenesis of IBD.

Regarding diet, it has been shown that diet can induce epigenetic changes related to IBD. The human diet is influenced by environmental and cultural practices and can influence intestinal inflammation through various pathways. They seem to be related to the alteration of the expression of miRNAs, the alteration of the composition of the intestinal microbiome and metabolome, and the influence of the integrity and permeability of the gastrointestinal barrier [173]. The elements of the Western diet, characterized by a low content of fiber, fruits, and vegetables, and a lack of micronutrients, as well as a diet high in fat, seem to be associated with epigenetic changes, such as a decrease in certain miRNAs (miR-143/145a, miR-148a, and miR-152) in colonocytes, increasing the aggravation of colitis [174]. Furthermore, a high-fat diet can modify the miRNA profile of visceral adipose exosomes towards a pro-inflammatory phenotype, predisposing the intestine to inflammation through the promotion of M1 macrophage polarization [175]. A diet high in fat has been associated with an increased risk of developing UC with a high intake of total fat, polyunsaturated fatty acids (PUFAs), and omega-6 fatty acids, as well as an increased risk of CD with a high intake of PUFAs, omega-6 fatty acids, and saturated fats [176]. From animal studies, the mechanism could be due to a possible increase in colonic epithelial natural killer T cells and a reduction in circulating Tregs [177]. In addition, a diet high in saturated fats can alter the composition of bile acids, which can favor the proliferation of sulfate-reducing bacteria, leading to an increase in hydrogen sulfide, which is toxic to the mucosa. A diet low or deficient in methyl donors can contribute to intestinal inflammation by reducing the expression of histone deacetylase (SIRT1). This reduction promotes endoplasmic reticulum stress and abnormal intestinal expression of CEA Cell Adhesion Molecule 6 (CEACAM6), facilitating the colonization of adherent-invasive *Escherichia coli* and subsequent inflammation [178].

**Figure 3:**
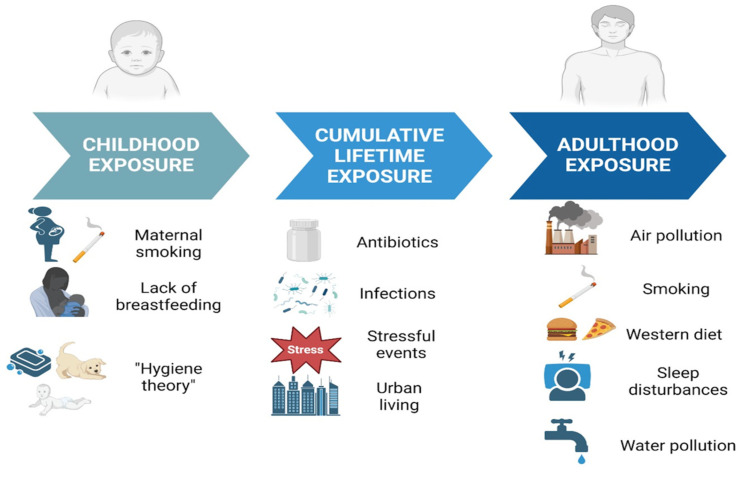
Environmental exposures contributing to IBD pathogenesis across the lifespan. The figure highlights risk factors at different life stages—childhood exposure, including maternal smoking, lack of breastfeeding, and reduced microbial diversity associated with the “hygiene theory”; cumulative lifetime exposure, such as antibiotic use, infections, stressful events, and urban living; and adulthood exposure, including air and water pollution, smoking, Western diets, and sleep disturbances. These exposures may influence immune responses and gut microbiome composition, increasing susceptibility to inflammation and IBD development.

Fewer data are available on the consumption of animal proteins and food additives. Some recent data report a greater exposure of CD patients to a larger number of processed foods and food additives compared to control groups, which could predispose to the development of CD and ongoing inflammation [179]. Food additives such as aluminum, titanium dioxide (TiO2), and microparticles/nanoparticles have been implicated in murine models of colitis [180,181]. TiO2 can be absorbed by intestinal epithelial cells and can exacerbate inflammation in predisposed individuals [179]. Furthermore, there are studies in the literature that have associated the consumption of meat with the risk of IBDs, although human studies in this area are still rather heterogeneous [182,183]. Regarding alcohol, chronic exposure can increase the expression of miR-122a and miR-155 in the intestine, decreasing the expression of occludins, leading to increased intestinal permeability, activation of TNF-α and NF-κB in the intestine, respectively [184]. In contrast, polyphenols, present mainly in fruits and vegetables [185,186,187], can reduce the risk of intestinal inflammation, mainly by modifying the miR profile and inhibiting HDACs. Furthermore, in humans, a high intake of dietary fiber, particularly from cruciferous fruits and vegetables, has been associated with a reduced risk of CD (HR = 0.59; 95% CI: 0.39–0.90) [188,189]. The protective effect of fiber was observed as statistically significant in those who consumed more than 22.1 g/day. A high intake of fruit has also been associated with a 73–80% reduction in the risk of CD in the same study [190]. The role of environmental exposures across childhood, cumulative lifetime, and adulthood in IBD pathogenesis is summarized in Figure 3.

## 6. Conclusions

Our findings from the most recent research confirm that IBDs are complex disorders driven by the intricate interplay between genetic predisposition, immune dysregulation, gut microbiota alterations, and environmental exposures. Although knowledge on this topic continues to grow, much remains to be understood. More in depth, the intersection of these pathogenic mechanisms and the relative contribution to disease onset and progression needs to be investigated. Future research is essential to further elucidate these dynamics and improve our understanding of IBD pathogenesis.

In the meantime, preventive strategies during pregnancy and early childhood offer significant potential. Maternal smoking, poor nutrition, and antibiotic exposure in childhood have been associated with increased IBD risk, whereas breastfeeding and diets rich in fiber and polyphenols provide protective effects by fostering healthy immune and microbiota development. These insights underscore the importance of addressing modifiable factors during these critical life stages.

Therapeutically, efforts to restore microbial diversity, repair epithelial barrier function, and modulate immune responses represent promising approaches. Advances in multi-omics technologies and exploration of neglected microbiome components, such as the virome and mycobiome, will further enhance our understanding and open new avenues for precision medicine. Thanks to these scientific advancements, the next objectives will focus on reducing the burden of IBD, developing effective and personalized treatments and minimizing adverse effects. The ultimate goal will be reducing the incidence of IBD by integrating early-life prevention strategies with innovative therapeutic approaches.

## References

[1] Zhang Y.-Z. (2014). Inflammatory Bowel Disease: Pathogenesis. World J. Gastroenterol..

[2] Abdulla M., Mohammed N. (2022). A Review on Inflammatory Bowel Diseases: Recent Molecular Pathophysiology Advances. Biol. Targets Ther..

[3] Annese V. (2020). Genetics and Epigenetics of IBD. Pharmacol. Res..

[4] Aden K., Reindl W. (2019). The Gut Microbiome in Inflammatory Bowel Diseases: Diagnostic and Therapeutic Implications. Visc. Med..

[5] Ho S.-M., Lewis J.D., Mayer E.A., Bernstein C.N., Plevy S.E., Chuang E., Rappaport S.M., Croitoru K., Korzenik J.R., Krischer J. (2019). Challenges in IBD Research: Environmental Triggers. Inflamm. Bowel Dis..

[6] Guan Q. (2019). A Comprehensive Review and Update on the Pathogenesis of Inflammatory Bowel Disease. J. Immunol. Res..

[7] Baumgart D.C., Carding S.R. (2007). Inflammatory Bowel Disease: Cause and Immunobiology. Lancet.

[8] Kong L., Pokatayev V., Lefkovith A., Carter G.T., Creasey E.A., Krishna C., Subramanian S., Kochar B., Ashenberg O., Lau H. (2023). The Landscape of Immune Dysregulation in Crohn’s Disease Revealed through Single-Cell Transcriptomic Profiling in the Ileum and Colon. Immunity.

[9] Okumura R., Takeda K. (2017). Roles of Intestinal Epithelial Cells in the Maintenance of Gut Homeostasis. Exp. Mol. Med..

[10] Kim Y.S., Ho S.B. (2010). Intestinal Goblet Cells and Mucins in Health and Disease: Recent Insights and Progress. Curr. Gastroenterol. Rep..

[11] Wei J., Chen C., Feng J., Zhou S., Feng X., Yang Z., Lu H., Tao H., Li L., Xv H. (2023). Muc2 Mucin O-Glycosylation Interacts with Enteropathogenic Escherichia Coli to Influence the Development of Ulcerative Colitis Based on the NF-kB Signaling Pathway. J. Transl. Med..

[12] Ashton J.J., Boukas K., Davies J., Stafford I.S., Vallejo A.F., Haggarty R., Coelho T.A.F., Batra A., Afzal N.A., Vadgama B. (2021). Ileal Transcriptomic Analysis in Paediatric Crohn’s Disease Reveals *IL17*- and *NOD*-Signalling Expression Signatures in Treatment-Naïve Patients and Identifies Epithelial Cells Driving Differentially Expressed Genes. J. Crohns Colitis.

[13] Bennstein S.B., Uhrberg M. (2024). Circulating Innate Lymphoid Cells (cILCs): Unconventional Lymphocytes with Hidden Talents. J. Allergy Clin. Immunol..

[14] Krämer B., Goeser F., Lutz P., Glässner A., Boesecke C., Schwarze-Zander C., Kaczmarek D., Nischalke H.D., Branchi V., Manekeller S. (2017). Compartment-Specific Distribution of Human Intestinal Innate Lymphoid Cells Is Altered in HIV Patients under Effective Therapy. PLoS Pathog..

[15] Srivastava R.K., Sapra L., Bhardwaj A., Mishra P.K., Verma B., Baig Z. (2023). Unravelling the Immunobiology of Innate Lymphoid Cells (ILCs): Implications in Health and Disease. Cytokine Growth Factor Rev..

[16] Bao B., Wang Y., Boudreau P., Song X., Wu M., Chen X., Patik I., Tang Y., Ouahed J., Ringel A. (2024). Bacterial Sphingolipids Exacerbate Colitis by Inhibiting ILC3-Derived IL-22 Production. Cell. Mol. Gastroenterol. Hepatol..

[17] Eken A., Singh A.K., Treuting P.M., Oukka M. (2014). IL-23R+ Innate Lymphoid Cells Induce Colitis via Interleukin-22-Dependent Mechanism. Mucosal Immunol..

[18] Mazzurana L., Bonfiglio F., Forkel M., D’Amato M., Halfvarson J., Mjösberg J. (2021). Crohn’s Disease Is Associated With Activation of Circulating Innate Lymphoid Cells. Inflamm. Bowel Dis..

[19] Hunter M.M., Wang A., Parhar K.S., Johnston M.J.G., Van Rooijen N., Beck P.L., McKay D.M. (2010). In Vitro-Derived Alternatively Activated Macrophages Reduce Colonic Inflammation in Mice. Gastroenterology.

[20] Zhang K., Guo J., Yan W., Xu L. (2023). Macrophage Polarization in Inflammatory Bowel Disease. Cell Commun. Signal..

[21] Lissner D., Schumann M., Batra A., Kredel L.-I., Kühl A.A., Erben U., May C., Schulzke J.-D., Siegmund B. (2015). Monocyte and M1 Macrophage-Induced Barrier Defect Contributes to Chronic Intestinal Inflammation in IBD. Inflamm. Bowel Dis..

[22] Ma S., Zhang J., Liu H., Li S., Wang Q. (2022). The Role of Tissue-Resident Macrophages in the Development and Treatment of Inflammatory Bowel Disease. Front. Cell Dev. Biol..

[23] Zhang M., Li X., Zhang Q., Yang J., Liu G. (2023). Roles of Macrophages on Ulcerative Colitis and Colitis-Associated Colorectal Cancer. Front. Immunol..

[24] Garrido-Trigo A., Corraliza A.M., Veny M., Dotti I., Melón-Ardanaz E., Rill A., Crowell H.L., Corbí Á., Gudiño V., Esteller M. (2023). Macrophage and Neutrophil Heterogeneity at Single-Cell Spatial Resolution in Human Inflammatory Bowel Disease. Nat. Commun..

[25] Azramezani Kopi T., Amini Kadijani A., Parsian H., Shahrokh S., Asadzadeh Aghdaei H., Mirzaei A., Balaii H., Zali M.R. (2019). The Value of mRNA Expression of S100A8 and S100A9 as Blood-Based Biomarkers of Inflammatory Bowel Disease. Arab J. Gastroenterol..

[26] Okada K., Itoh H., Ikemoto M. (2020). Circulating S100A8/A9 Is Potentially a Biomarker That Could Reflect the Severity of Experimental Colitis in Rats. Heliyon.

[27] Luo X., Wang R., Zhang X., Wen X., Deng S., Xie W. (2023). Identification CCL2,CXCR2,S100A9 of the Immune-Related Gene Markers and Immune Infiltration Characteristics of Inflammatory Bowel Disease and Heart Failure via Bioinformatics Analysis and Machine Learning. Front. Cardiovasc. Med..

[28] Poggi A., Benelli R., Venè R., Costa D., Ferrari N., Tosetti F., Zocchi M.R. (2019). Human Gut-Associated Natural Killer Cells in Health and Disease. Front. Immunol..

[29] Zaiatz Bittencourt V., Jones F., Tosetto M., Doherty G.A., Ryan E.J. (2021). Dysregulation of Metabolic Pathways in Circulating Natural Killer Cells Isolated from Inflammatory Bowel Disease Patients. J. Crohns Colitis.

[30] Geremia A., Biancheri P., Allan P., Corazza G.R., Di Sabatino A. (2014). Innate and Adaptive Immunity in Inflammatory Bowel Disease. Autoimmun. Rev..

[31] Kałużna A., Olczyk P., Komosińska-Vassev K. (2022). The Role of Innate and Adaptive Immune Cells in the Pathogenesis and Development of the Inflammatory Response in Ulcerative Colitis. J. Clin. Med..

[32] Mitsialis V., Wall S., Liu P., Ordovas-Montanes J., Parmet T., Vukovic M., Spencer D., Field M., McCourt C., Toothaker J. (2020). Single-Cell Analyses of Colon and Blood Reveal Distinct Immune Cell Signatures of Ulcerative Colitis and Crohn’s Disease. Gastroenterology.

[33] Medina T.S., Murison A., Smith M., Kinker G.S., Chakravarthy A., Vitiello G.A.F., Turpin W., Shen S.Y., Yau H.L., Sarmento O.F. (2023). The Chromatin and Single-Cell Transcriptional Landscapes of CD4 T Cells in Inflammatory Bowel Disease Link Risk Loci with a Proinflammatory Th17 Cell Population. Front. Immunol..

[34] Cao M., Chen P., Peng B., Cheng Y., Xie J., Hou Z., Chen H., Ye L., Li H., Wang H. (2023). The Transcription Factor ELF4 Alleviates Inflammatory Bowel Disease by Activating IL1RN Transcription, Suppressing Inflammatory TH17 Cell Activity, and Inducing Macrophage M2 Polarization. Front. Immunol..

[35] Tanemoto S., Sujino T., Miyamoto K., Moody J., Yoshimatsu Y., Ando Y., Koya I., Harada Y., Tojo A.O., Ono K. (2022). Single-Cell Transcriptomics of Human Gut T Cells Identifies Cytotoxic CD4^+^CD8A^+^ T Cells Related to Mouse CD4 Cytotoxic T Cells. Front. Immunol..

[36] Casalegno Garduño R., Däbritz J. (2021). New Insights on CD8^+^ T Cells in Inflammatory Bowel Disease and Therapeutic Approaches. Front. Immunol..

[37] Corridoni D., Antanaviciute A., Gupta T., Fawkner-Corbett D., Aulicino A., Jagielowicz M., Parikh K., Repapi E., Taylor S., Ishikawa D. (2020). Single-Cell Atlas of Colonic CD8^+^ T Cells in Ulcerative Colitis. Nat. Med..

[38] Globig A.-M., Mayer L.S., Heeg M., Andrieux G., Ku M., Otto-Mora P., Hipp A.V., Zoldan K., Pattekar A., Rana N. (2022). Exhaustion of CD39-Expressing CD8^+^ T Cells in Crohn’s Disease Is Linked to Clinical Outcome. Gastroenterology.

[39] Mayne C.G., Williams C.B. (2013). Induced and Natural Regulatory T Cells in the Development of Inflammatory Bowel Disease: *Inflamm*. Bowel Dis..

[40] Yan J., Zeng Y., Guan Z., Li Z., Luo S., Niu J., Zhao J., Gong H., Huang T., Li Z. (2024). Inherent Preference for Polyunsaturated Fatty Acids Instigates Ferroptosis of Treg Cells That Aggravates High-Fat-Diet-Related Colitis. Cell Rep..

[41] Boland B.S., He Z., Tsai M.S., Olvera J.G., Omilusik K.D., Duong H.G., Kim E.S., Limary A.E., Jin W., Milner J.J. (2020). Heterogeneity and Clonal Relationships of Adaptive Immune Cells in Ulcerative Colitis Revealed by Single-Cell Analyses. Sci. Immunol..

[42] Zhang Q., Zeng Z., Wei N., Su Y., Wang J., Ni Q., Wang Y., Yang J., Liu X., Xu H. (2024). Mesenteric Lymph Nodes: A Critical Site for the up-Regulatory Effect of hUC-MSCs on Treg Cells by Producing TGF-β1 in Colitis Treatment. Stem Cell Res. Ther..

[43] Eiro N., Fraile M., González-Jubete A., González L.O., Vizoso F.J. (2022). Mesenchymal (Stem) Stromal Cells Based as New Therapeutic Alternative in Inflammatory Bowel Disease: Basic Mechanisms, Experimental and Clinical Evidence, and Challenges. Int. J. Mol. Sci..

[44] Stavely R., Robinson A.M., Fraser S., Filippone R.T., Stojanovska V., Eri R., Apostolopoulos V., Sakkal S., Nurgali K. (2024). Bone Marrow-Derived Mesenchymal Stem Cells Mitigate Chronic Colitis and Enteric Neuropathy via Anti-Inflammatory and Anti-Oxidative Mechanisms. Sci. Rep..

[45] Dave M., Dev A., Somoza R.A., Zhao N., Viswanath S., Mina P.R., Chirra P., Obmann V.C., Mahabeleshwar G.H., Menghini P. (2024). MSCs Mediate Long-Term Efficacy in a Crohn’s Disease Model by Sustained Anti-Inflammatory Macrophage Programming via Efferocytosis. npj Regen. Med..

[46] Scanu M., Toto F., Petito V., Masi L., Fidaleo M., Puca P., Baldelli V., Reddel S., Vernocchi P., Pani G. (2024). An Integrative Multi-Omic Analysis Defines Gut Microbiota, Mycobiota, and Metabolic Fingerprints in Ulcerative Colitis Patients. Front. Cell. Infect. Microbiol..

[47] Luchen C.C., Chibuye M., Spijker R., Simuyandi M., Chisenga C., Bosomprah S., Chilengi R., Schultsz C., Mende D.R., Harris V.C. (2023). Impact of Antibiotics on Gut Microbiome Composition and Resistome in the First Years of Life in Low- to Middle-Income Countries: A Systematic Review. PLoS Med..

[48] Pan R., He Y., Yuan J., Zhao S., Ma M., Chai Z., Ji X., Hu X., He C., Zhou D. (2024). The Role of Antibiotic Exposure as Risk Factor for IBD Epidemic: An Updated Meta-Analysis. J. Gastroenterol. Hepatol..

[49] Mårild K., Lerchova T., Östensson M., Imberg H., Størdal K., Ludvigsson J. (2024). Early-Life Infections, Antibiotics and Later Risk of Childhood and Early Adult-Onset Inflammatory Bowel Disease: Pooled Analysis of Two Scandinavian Birth Cohorts. Aliment. Pharmacol. Ther..

[50] Pascal V., Pozuelo M., Borruel N., Casellas F., Campos D., Santiago A., Martinez X., Varela E., Sarrabayrouse G., Machiels K. (2017). A Microbial Signature for Crohn’s Disease. Gut.

[51] Abdel-Rahman L.I.H., Morgan X.C. (2023). Searching for a Consensus Among Inflammatory Bowel Disease Studies: A Systematic Meta-Analysis. Inflamm. Bowel Dis..

[52] Alam M.T., Amos G.C.A., Murphy A.R.J., Murch S., Wellington E.M.H., Arasaradnam R.P. (2020). Microbial Imbalance in Inflammatory Bowel Disease Patients at Different Taxonomic Levels. Gut Pathog..

[53] Amos G.C.A., Sergaki C., Logan A., Iriarte R., Bannaga A., Chandrapalan S., Wellington E.M.H., Rijpkema S., Arasaradnam R.P. (2021). Exploring How Microbiome Signatures Change across Inflammatory Bowel Disease Conditions and Disease Locations. Sci. Rep..

[54] Britton G.J., Contijoch E.J., Mogno I., Vennaro O.H., Llewellyn S.R., Ng R., Li Z., Mortha A., Merad M., Das A. (2019). Microbiotas from Humans with Inflammatory Bowel Disease Alter the Balance of Gut Th17 and RORγt^+^ Regulatory T Cells and Exacerbate Colitis in Mice. Immunity.

[55] Sugihara K., Kamada N. (2024). Metabolic Network of the Gut Microbiota in Inflammatory Bowel Disease. Inflamm. Regen..

[56] Dong D., Su T., Chen W., Wang D., Xue Y., Lu Q., Jiang C., Ni Q., Mao E., Peng Y. (2023). *Clostridioides Difficile* Aggravates Dextran Sulfate Solution (DSS)-Induced Colitis by Shaping the Gut Microbiota and Promoting Neutrophil Recruitment. Gut Microbes.

[57] Leon-Coria A., Kumar M., Workentine M., Moreau F., Surette M., Chadee K. (2021). Muc2 Mucin and Nonmucin Microbiota Confer Distinct Innate Host Defense in Disease Susceptibility and Colonic Injury. Cell. Mol. Gastroenterol. Hepatol..

[58] Li M., Ding Y., Wei J., Dong Y., Wang J., Dai X., Yan J., Chu F., Zhang K., Meng F. (2024). Gut Microbiota Metabolite Indole-3-Acetic Acid Maintains Intestinal Epithelial Homeostasis Through Mucin Sulfation. Gut Microbes.

[59] Leibovitzh H., Lee S.-H., Xue M., Raygoza Garay J.A., Hernandez-Rocha C., Madsen K.L., Meddings J.B., Guttman D.S., Espin-Garcia O., Smith M.I. (2022). Altered Gut Microbiome Composition and Function Are Associated with Gut Barrier Dysfunction in Healthy Relatives of Patients With Crohn’s Disease. Gastroenterology.

[60] Luchetti M.M., Ciccia F., Avellini C., Benfaremo D., Rizzo A., Spadoni T., Svegliati S., Marzioni D., Santinelli A., Costantini A. (2021). Gut Epithelial Impairment, Microbial Translocation and Immune System Activation in Inflammatory Bowel Disease–Associated Spondyloarthritis. Rheumatology.

[61] Alexander K.L., Zhao Q., Reif M., Rosenberg A.F., Mannon P.J., Duck L.W., Elson C.O. (2021). Human Microbiota Flagellins Drive Adaptive Immune Responses in Crohn’s Disease. Gastroenterology.

[62] Vujkovic-Cvijin I., Welles H.C., Ha C.W.Y., Huq L., Mistry S., Brenchley J.M., Trinchieri G., Devkota S., Belkaid Y. (2022). The Systemic Anti-Microbiota IgG Repertoire Can Identify Gut Bacteria That Translocate Across Gut Barrier Surfaces. Sci. Transl. Med..

[63] Gallagher K., Catesson A., Griffin J.L., Holmes E., Williams H.R.T. (2021). Metabolomic Analysis in Inflammatory Bowel Disease: A Systematic Review. J. Crohns Colitis.

[64] Ning L., Zhou Y.-L., Sun H., Zhang Y., Shen C., Wang Z., Xuan B., Zhao Y., Ma Y., Yan Y. (2023). Microbiome and Metabolome Features in Inflammatory Bowel Disease via Multi-Omics Integration Analyses across Cohorts. Nat. Commun..

[65] Zhao H., Zhou Y., Xu J., Zhang Y., Wang H., Zhao C., Huang H., Yang J., Huang C., Li Y. (2024). Short-Chain Fatty Acid-Producing Bacterial Strains Attenuate Experimental Ulcerative Colitis by Promoting M2 Macrophage Polarization via JAK/STAT3/FOXO3 Axis Inactivation. J. Transl. Med..

[66] Tian X., Li S., Wang C., Zhang Y., Feng X., Yan Q., Guo R., Wu F., Wu C., Wang Y. (2024). Gut Virome-Wide Association Analysis Identifies Cross-Population Viral Signatures for Inflammatory Bowel Disease. Microbiome.

[67] Ungaro F., Massimino L., Furfaro F., Rimoldi V., Peyrin-Biroulet L., D’Alessio S., Danese S. (2019). Metagenomic Analysis of Intestinal Mucosa Revealed a Specific Eukaryotic Gut Virome Signature in Early-Diagnosed Inflammatory Bowel Disease. Gut Microbes.

[68] Bernardi F., Ungaro F., D’Amico F., Zilli A., Parigi T.L., Massimino L., Allocca M., Danese S., Furfaro F. (2024). The Role of Viruses in the Pathogenesis of Immune-Mediated Gastro-Intestinal Diseases. Int. J. Mol. Sci..

[69] Carasso S., Zaatry R., Hajjo H., Kadosh-Kariti D., Ben-Assa N., Naddaf R., Mandelbaum N., Pressman S., Chowers Y., Gefen T. (2024). Inflammation and Bacteriophages Affect DNA Inversion States and Functionality of the Gut Microbiota. Cell Host Microbe.

[70] Yu M., Ding H., Gong S., Luo Y., Lin H., Mu Y., Li H., Li X., Zhong M. (2023). Fungal Dysbiosis Facilitates Inflammatory Bowel Disease by Enhancing CD4^+^ T Cell Glutaminolysis. Front. Cell. Infect. Microbiol..

[71] Iliev I.D., Funari V.A., Taylor K.D., Nguyen Q., Reyes C.N., Strom S.P., Brown J., Becker C.A., Fleshner P.R., Dubinsky M. (2012). Interactions Between Commensal Fungi and the C-Type Lectin Receptor Dectin-1 Influence Colitis. Science.

[72] Zhang W., Lyu M., Bessman N.J., Xie Z., Arifuzzaman M., Yano H., Parkhurst C.N., Chu C., Zhou L., Putzel G.G. (2022). Gut-Innervating Nociceptors Regulate the Intestinal Microbiota to Promote Tissue Protection. Cell.

[73] Lopetuso L.R., Deleu S., Godny L., Petito V., Puca P., Facciotti F., Sokol H., Ianiro G., Masucci L., Abreu M. (2023). The First International Rome Consensus Conference on Gut Microbiota and Faecal Microbiota Transplantation in Inflammatory Bowel Disease. Gut.

[74] Costello S.P., Soo W., Bryant R.V., Jairath V., Hart A.L., Andrews J.M. (2017). Systematic Review with Meta-Analysis: Faecal Microbiota Transplantation for the Induction of Remission for Active Ulcerative Colitis. Aliment. Pharmacol. Ther..

[75] Caldeira L.d.F., Borba H.H., Tonin F.S., Wiens A., Fernandez-Llimos F., Pontarolo R. (2020). Fecal Microbiota Transplantation in Inflammatory Bowel Disease Patients: A Systematic Review and Meta-Analysis. PLoS ONE.

[76] Loddo I., Romano C. (2015). Inflammatory Bowel Disease: Genetics, Epigenetics, and Pathogenesis. Front. Immunol..

[77] Liu T.-C., Stappenbeck T.S. (2016). Genetics and Pathogenesis of Inflammatory Bowel Disease. Annu. Rev. Pathol. Mech. Dis..

[78] Palmieri O., Latiano A., Valvano R., D’Incà R., Vecchi M., Sturniolo G.C., Saibeni S., Peyvandi F., Bossa F., Zagaria C. (2006). Variants of OCTN1–2 Cation Transporter Genes Are Associated with Both Crohn’s Disease and Ulcerative Colitis. Aliment. Pharmacol. Ther..

[79] Hu S., Vich Vila A., Gacesa R., Collij V., Stevens C., Fu J.M., Wong I., Talkowski M.E., Rivas M.A., Imhann F. (2020). Whole Exome Sequencing Analyses Reveal Gene–Microbiota Interactions in the Context of IBD. Gut.

[80] Galluccio M., Tripicchio M., Pochini L. (2024). The Human OCTN Sub-Family: Gene and Protein Structure, Expression, and Regulation. Int. J. Mol. Sci..

[81] Del Chierico F., Masi L., Petito V., Baldelli V., Puca P., Benvenuto R., Fidaleo M., Palucci I., Lopetuso L.R., Caristo M.E. (2024). Solute Transporter OCTN1/Slc22a4 Affects Disease Severity and Response to Infliximab in Experimental Colitis: Role of Gut Microbiota and Immune Modulation. Inflamm. Bowel Dis..

[82] Luo P., Yang Z., Chen B., Zhong X. (2020). The Multifaceted Role of CARD9 in Inflammatory Bowel Disease. J. Cell. Mol. Med..

[83] Danne C., Michaudel C., Skerniskyte J., Planchais J., Magniez A., Agus A., Michel M.-L., Lamas B., Da Costa G., Spatz M. (2023). CARD9 in Neutrophils Protects from Colitis and Controls Mitochondrial Metabolism and Cell Survival. Gut.

[84] Danne C., Lamas B., Lavelle A., Michel M.-L., Da Costa G., Pham H.-P., Lefevre A., Bridonneau C., Bredon M., Planchais J. (2024). Dissecting the Respective Roles of Microbiota and Host Genetics in the Susceptibility of *Card9*^−/−^ Mice to Colitis. Microbiome.

[85] Valatas V., Kolios G., Bamias G. (2019). TL1A (TNFSF15) and DR3 (TNFRSF25): A Co-Stimulatory System of Cytokines With Diverse Functions in Gut Mucosal Immunity. Front. Immunol..

[86] Sun R., Hedl M., Abraham C. (2021). TNFSF15 Promotes Antimicrobial Pathways in Human Macrophages and These Are Modulated by TNFSF15 Disease-Risk Variants. Cell. Mol. Gastroenterol. Hepatol..

[87] Kakuta Y., Ichikawa R., Fuyuno Y., Hirano A., Umeno J., Torisu T., Watanabe K., Asakura A., Nakano T., Izumiyama Y. (2020). An Integrated Genomic and Transcriptomic Analysis Reveals Candidates of Susceptibility Genes for Crohn’s Disease in Japanese Populations. Sci. Rep..

[88] Zhou Y., Zhu Y., Jiang H., Chen Z., Lu B., Li J., Shen X. (2020). Polymorphism Rs6478109 in the *TNFSF15* Gene Contributes to the Susceptibility to Crohn’s Disease but Not Ulcerative Colitis: A Meta-Analysis. J. Int. Med. Res..

[89] Muise A.M., Snapper S.B., Kugathasan S. (2012). The Age of Gene Discovery in Very Early Onset Inflammatory Bowel Disease. Gastroenterology.

[90] Uhlig H.H., Schwerd T., Koletzko S., Shah N., Kammermeier J., Elkadri A., Ouahed J., Wilson D.C., Travis S.P., Turner D. (2014). The Diagnostic Approach to Monogenic Very Early Onset Inflammatory Bowel Disease. Gastroenterology.

[91] Nambu R., Warner N., Mulder D.J., Kotlarz D., McGovern D.P.B., Cho J., Klein C., Snapper S.B., Griffiths A.M., Iwama I. (2022). A Systematic Review of Monogenic Inflammatory Bowel Disease. Clin. Gastroenterol. Hepatol..

[92] Arnadottir G.A., Norddahl G.L., Gudmundsdottir S., Agustsdottir A.B., Sigurdsson S., Jensson B.O., Bjarnadottir K., Theodors F., Benonisdottir S., Ivarsdottir E.V. (2018). A Homozygous Loss-of-Function Mutation Leading to CYBC1 Deficiency Causes Chronic Granulomatous Disease. Nat. Commun..

[93] Abdel-Motal U.M., Hubrack S.Z., Bullock A.N., Al-Marri A.A., Agrebi N., Al-Subaiey A.A., Ibrahim N.A., Charles A.K., Al-Kaabi S.R., Al-Mohannadi M.J. (2021). Human AGR2 Deficiency Causes Mucus Barrier Dysfunction and Infantile Inflammatory Bowel Disease. Cell. Mol. Gastroenterol. Hepatol..

[94] Neehus A.-L., Moriya K., Nieto-Patlán A., Le Voyer T., Lévy R., Özen A., Karakoc-Aydiner E., Baris S., Yildiran A., Altundag E. (2021). Impaired Respiratory Burst Contributes to Infections in PKCδ-Deficient Patients. J. Exp. Med..

[95] Ozen A., Comrie W.A., Ardy R.C., Conde C.D., Dalgic B., Beser F., Morawski A.R., Karakoc-Aydiner E., Tutar E., Baris S. (2017). CD55 Deficiency, Early-Onset Protein-Losing Enteropathy, and Thrombosis. N. Engl. J. Med..

[96] Azabdaftari A., Jones K.D.J., Kammermeier J., Uhlig H.H. (2023). Monogenic Inflammatory Bowel Disease-Genetic Variants, Functional Mechanisms and Personalised Medicine in Clinical Practice. Hum. Genet..

[97] Sudhakar P., Andrighetti T., Verstockt S., Caenepeel C., Ferrante M., Sabino J., Verstockt B., Vermeire S. (2022). Integrated Analysis of Microbe-Host Interactions in Crohn’s Disease Reveals Potential Mechanisms of Microbial Proteins on Host Gene Expression. iScience.

[98] Ng S.C., Bernstein C.N., Vatn M.H., Lakatos P.L., Loftus E.V., Tysk C., O’Morain C., Moum B., Colombel J.-F. (2013). Epidemiology and Natural History Task Force of the International Organization of Inflammatory Bowel Disease (IOIBD) Geographical Variability and Environmental Risk Factors in Inflammatory Bowel Disease. Gut.

[99] Mahid S.S., Minor K.S., Soto R.E., Hornung C.A., Galandiuk S. (2006). Smoking and Inflammatory Bowel Disease: A Meta-Analysis. Mayo Clin. Proc..

[100] Dunford A.R., Sangster J.M. (2017). Maternal and Paternal Periconceptional Nutrition as an Indicator of Offspring Metabolic Syndrome Risk in Later Life through Epigenetic Imprinting: A Systematic Review. Diabetes Metab. Syndr..

[101] Zong D., Liu X., Li J., Ouyang R., Chen P. (2019). The Role of Cigarette Smoke-Induced Epigenetic Alterations in Inflammation. Epigenet. Chromatin.

[102] Marciniak A., Patro-Małysza J., Kimber-Trojnar Ż., Marciniak B., Oleszczuk J., Leszczyńska-Gorzelak B. (2017). Fetal Programming of the Metabolic Syndrome. Taiwan. J. Obstet. Gynecol..

[103] Grossniklaus U., Kelly W.G., Kelly B., Ferguson-Smith A.C., Pembrey M., Lindquist S. (2013). Transgenerational Epigenetic Inheritance: How Important Is It?. Nat. Rev. Genet..

[104] Ventham N.T., Kennedy N.A., Nimmo E.R., Satsangi J. (2013). Beyond Gene Discovery in Inflammatory Bowel Disease: The Emerging Role of Epigenetics. Gastroenterology.

[105] Schaible T.D., Harris R.A., Dowd S.E., Smith C.W., Kellermayer R. (2011). Maternal Methyl-Donor Supplementation Induces Prolonged Murine Offspring Colitis Susceptibility in Association with Mucosal Epigenetic and Microbiomic Changes. Hum. Mol. Genet..

[106] Lim A.I., McFadden T., Link V.M., Han S.-J., Karlsson R.-M., Stacy A., Farley T.K., Lima-Junior D.S., Harrison O.J., Desai J.V. (2021). Prenatal Maternal Infection Promotes Tissue-Specific Immunity and Inflammation in Offspring. Science.

[107] Van Der Sloot K.W.J., Weersma R.K., Alizadeh B.Z., Dijkstra G. (2020). Identification of Environmental Risk Factors Associated With the Development of Inflammatory Bowel Disease. J. Crohns Colitis.

[108] Wiklund P., Karhunen V., Richmond R.C., Parmar P., Rodriguez A., De Silva M., Wielscher M., Rezwan F.I., Richardson T.G., Veijola J. (2019). DNA Methylation Links Prenatal Smoking Exposure to Later Life Health Outcomes in Offspring. Clin. Epigenet..

[109] Fragou D., Pakkidi E., Aschner M., Samanidou V., Kovatsi L. (2019). Smoking and DNA Methylation: Correlation of Methylation with Smoking Behavior and Association with Diseases and Fetus Development Following Prenatal Exposure. Food Chem. Toxicol..

[110] McLean C., Jun S., Kozyrskyj A. (2019). Impact of Maternal Smoking on the Infant Gut Microbiota and Its Association with Child Overweight: A Scoping Review. World J. Pediatr..

[111] Xu L., Lochhead P., Ko Y., Claggett B., Leong R.W., Ananthakrishnan A.N. (2017). Systematic Review with Meta-Analysis: Breastfeeding and the Risk of Crohn’s Disease and Ulcerative Colitis. Aliment. Pharmacol. Ther..

[112] Klement E., Cohen R.V., Boxman J., Joseph A., Reif S. (2004). Breastfeeding and Risk of Inflammatory Bowel Disease: A Systematic Review with Meta-Analysis. Am. J. Clin. Nutr..

[113] Gordon H., Blad W., Trier Møller F., Orchard T., Steel A., Trevelyan G., Ng S., Harbord M. (2022). UK IBD Twin Registry: Concordance and Environmental Risk Factors of Twins with IBD. Dig. Dis. Sci..

[114] Penders J., Thijs C., van den Brandt P.A., Kummeling I., Snijders B., Stelma F., Adams H., van Ree R., Stobberingh E.E. (2007). Gut Microbiota Composition and Development of Atopic Manifestations in Infancy: The KOALA Birth Cohort Study. Gut.

[115] M’Rabet L., Vos A.P., Boehm G., Garssen J. (2008). Breast-Feeding and Its Role in Early Development of the Immune System in Infants: Consequences for Health Later in Life1. J. Nutr..

[116] Keag O.E., Norman J.E., Stock S.J. (2018). Long-Term Risks and Benefits Associated with Cesarean Delivery for Mother, Baby, and Subsequent Pregnancies: Systematic Review and Meta-Analysis. PLoS Med..

[117] Li Y., Tian Y., Zhu W., Gong J., Gu L., Zhang W., Guo Z., Li N., Li J. (2014). Cesarean Delivery and Risk of Inflammatory Bowel Disease: A Systematic Review and Meta-Analysis. Scand. J. Gastroenterol..

[118] Bruce A., Black M., Bhattacharya S. (2014). Mode of Delivery and Risk of Inflammatory Bowel Disease in the Offspring: Systematic Review and Meta-Analysis of Observational Studies. Inflamm. Bowel Dis..

[119] Kostic A.D., Xavier R.J., Gevers D. (2014). The Microbiome in Inflammatory Bowel Disease: Current Status and the Future Ahead. Gastroenterology.

[120] Dethlefsen L., Huse S., Sogin M.L., Relman D.A. (2008). The Pervasive Effects of an Antibiotic on the Human Gut Microbiota, as Revealed by Deep 16S rRNA Sequencing. PLoS Biol..

[121] Pérez-Cobas A.E., Gosalbes M.J., Friedrichs A., Knecht H., Artacho A., Eismann K., Otto W., Rojo D., Bargiela R., von Bergen M. (2013). Gut Microbiota Disturbance during Antibiotic Therapy: A Multi-Omic Approach. Gut.

[122] Ungaro R., Bernstein C.N., Gearry R., Hviid A., Kolho K.-L., Kronman M.P., Shaw S., Van Kruiningen H., Colombel J.-F., Atreja A. (2014). Antibiotics Associated with Increased Risk of New-Onset Crohn’s Disease but Not Ulcerative Colitis: A Meta-Analysis. Am. J. Gastroenterol..

[123] Manichanh C., Borruel N., Casellas F., Guarner F. (2012). The Gut Microbiota in IBD. Nat. Rev. Gastroenterol. Hepatol..

[124] Gevers D., Kugathasan S., Denson L.A., Vázquez-Baeza Y., Van Treuren W., Ren B., Schwager E., Knights D., Song S.J., Yassour M. (2014). The Treatment-Naive Microbiome in New-Onset Crohn’s Disease. Cell Host Microbe.

[125] Cholapranee A., Ananthakrishnan A.N. (2016). Environmental Hygiene and Risk of Inflammatory Bowel Diseases: A Systematic Review and Meta-Analysis. Inflamm. Bowel Dis..

[126] Michaux M., Chan J.M., Bergmann L., Chaves L.F., Klinkenberg B., Jacobson K. (2023). Spatial Cluster Mapping and Environmental Modeling in Pediatric Inflammatory Bowel Disease. World J. Gastroenterol..

[127] Song C., Yang J., Ye W., Zhang Y., Tang C., Li X., Zhou X., Xie Y. (2019). Urban-Rural Environmental Exposure during Childhood and Subsequent Risk of Inflammatory Bowel Disease: A Meta-Analysis. Expert Rev. Gastroenterol. Hepatol..

[128] Wu X.-W., Ji H.-Z., Yang M.-F., Wu L., Wang F.-Y. (2015). Helicobacter Pylori Infection and Inflammatory Bowel Disease in Asians: A Meta-Analysis. World J. Gastroenterol..

[129] Castaño-Rodríguez N., Kaakoush N.O., Lee W.S., Mitchell H.M. (2017). Dual Role of Helicobacter and Campylobacter Species in IBD: A Systematic Review and Meta-Analysis. Gut.

[130] Luther J., Dave M., Higgins P.D.R., Kao J.Y. (2010). Association between Helicobacter Pylori Infection and Inflammatory Bowel Disease: A Meta-Analysis and Systematic Review of the Literature. Inflamm. Bowel Dis..

[131] Rokkas T., Gisbert J.P., Niv Y., O’Morain C. (2015). The Association between Helicobacter Pylori Infection and Inflammatory Bowel Disease Based on Meta-Analysis. United Eur. Gastroenterol. J..

[132] Codolo G., Mazzi P., Amedei A., Del Prete G., Berton G., D’Elios M.M., de Bernard M. (2008). The Neutrophil-Activating Protein of Helicobacter Pylori down-Modulates Th2 Inflammation in Ovalbumin-Induced Allergic Asthma. Cell. Microbiol..

[133] Luther J., Owyang S.Y., Takeuchi T., Cole T.S., Zhang M., Liu M., Erb-Downward J., Rubenstein J.H., Chen C.-C., Pierzchala A.V. (2011). Helicobacter Pylori DNA Decreases Pro-Inflammatory Cytokine Production by Dendritic Cells and Attenuates Dextran Sodium Sulphate-Induced Colitis. Gut.

[134] Arnold I.C., Hitzler I., Müller A. (2012). The Immunomodulatory Properties of Helicobacter Pylori Confer Protection against Allergic and Chronic Inflammatory Disorders. Front. Cell. Infect. Microbiol..

[135] Okada H., Kuhn C., Feillet H., Bach J.-F. (2010). The “hygiene Hypothesis” for Autoimmune and Allergic Diseases: An Update. Clin. Exp. Immunol..

[136] Schaub B., Liu J., Höppler S., Schleich I., Huehn J., Olek S., Wieczorek G., Illi S., von Mutius E. (2009). Maternal Farm Exposure Modulates Neonatal Immune Mechanisms through Regulatory T Cells. J. Allergy Clin. Immunol..

[137] Hasosah M., Alhashmi W., Abualsaud R., Alamoudi A., Aljawad A., Tunkar M., Felemban N., Basalim A., Khan M., Alanazi A. (2022). Environmental Risk Factors for Childhood Inflammatory Bowel Diseases: A Multicenter Case-Control Study. Children.

[138] Strisciuglio C., Giugliano F., Martinelli M., Cenni S., Greco L., Staiano A., Miele E. (2017). Impact of Environmental and Familial Factors in a Cohort of Pediatric Patients With Inflammatory Bowel Disease. J. Pediatr. Gastroenterol. Nutr..

[139] Piovani D., Danese S., Peyrin-Biroulet L., Nikolopoulos G.K., Lytras T., Bonovas S. (2019). Environmental Risk Factors for Inflammatory Bowel Diseases: An Umbrella Review of Meta-Analyses. Gastroenterology.

[140] Jakobsen C., Paerregaard A., Munkholm P., Wewer V. (2013). Environmental Factors and Risk of Developing Paediatric Inflammatory Bowel Disease—A Population Based Study 2007–2009. J. Crohns Colitis.

[141] Sidik S. (2023). Chronic Stress Can Inflame the Gut—Now Scientists Know Why. Nature.

[142] Schneider K.M., Blank N., Alvarez Y., Thum K., Lundgren P., Litichevskiy L., Sleeman M., Bahnsen K., Kim J., Kardo S. (2023). The Enteric Nervous System Relays Psychological Stress to Intestinal Inflammation. Cell.

[143] Bayrer J.R., Castro J., Venkataraman A., Touhara K.K., Rossen N.D., Morrie R.D., Maddern J., Hendry A., Braverman K.N., Garcia-Caraballo S. (2023). Gut Enterochromaffin Cells Drive Visceral Pain and Anxiety. Nature.

[144] Karl J.P., Hatch A.M., Arcidiacono S.M., Pearce S.C., Pantoja-Feliciano I.G., Doherty L.A., Soares J.W. (2018). Effects of Psychological, Environmental and Physical Stressors on the Gut Microbiota. Front. Microbiol..

[145] Oligschlaeger Y., Yadati T., Houben T., Condello Oliván C.M., Shiri-Sverdlov R. (2019). Inflammatory Bowel Disease: A Stressed “Gut/Feeling”. Cells.

[146] Konturek P.C., Brzozowski T., Konturek S.J. (2011). Stress and the Gut: Pathophysiology, Clinical Consequences, Diagnostic Approach and Treatment Options. J. Physiol. Pharmacol..

[147] Hu D., Ren J., Wang G., Gu G., Liu S., Wu X., Chen J., Ren H., Hong Z., Li J. (2014). Geographic Mapping of Crohn’s Disease and Its Relation to Affluence in Jiangsu Province, an Eastern Coastal Province of China. Gastroenterol. Res. Pract..

[148] Kaplan G.G., Hubbard J., Korzenik J., Sands B.E., Panaccione R., Ghosh S., Wheeler A.J., Villeneuve P.J. (2010). The Inflammatory Bowel Diseases and Ambient Air Pollution: A Novel Association. Am. J. Gastroenterol..

[149] Sahoo D.K., Heilmann R.M., Paital B., Patel A., Yadav V.K., Wong D., Jergens A.E. (2023). Oxidative Stress, Hormones, and Effects of Natural Antioxidants on Intestinal Inflammation in Inflammatory Bowel Disease. Front. Endocrinol..

[150] Tian T., Wang Z., Zhang J. (2017). Pathomechanisms of Oxidative Stress in Inflammatory Bowel Disease and Potential Antioxidant Therapies. Oxid. Med. Cell. Longev..

[151] Qin J., Xia W., Liang G., Xu S., Zhao X., Wang D., Sun X., Li Y., Liu H. (2021). Association of Fine Particulate Matter with Glucose and Lipid Metabolism: A Longitudinal Study in Young Adults. Occup. Environ. Med..

[152] Rivas-Arancibia S., Miranda-Martínez A., Rodríguez-Martínez E., Hernández-Orozco E., Valdés-Fuentes M., De La Rosa-Sierra R. (2023). Ozone Environmental Pollution: Relationship between the Intestine and Neurodegenerative Diseases. Antioxidants.

[153] Ananthakrishnan A.N., McGinley E.L., Binion D.G., Saeian K. (2011). Ambient Air Pollution Correlates with Hospitalizations for Inflammatory Bowel Disease: An Ecologic Analysis. Inflamm. Bowel Dis..

[154] Adami G., Pontalti M., Cattani G., Rossini M., Viapiana O., Orsolini G., Benini C., Bertoldo E., Fracassi E., Gatti D. (2022). Association between Long-Term Exposure to Air Pollution and Immune-Mediated Diseases: A Population-Based Cohort Study. RMD Open.

[155] Liu Y., Wang T., Si B., Du H., Liu Y., Waqas A., Huang S., Zhao G., Chen S., Xu A. (2021). Intratracheally Instillated Diesel PM_2.5_ Significantly Altered the Structure and Composition of Indigenous Murine Gut Microbiota. Ecotoxicol. Environ. Saf..

[156] Okafor P.N., Dahlen A., Youssef M., Olayode A., Sonu I., Neshatian L., Nguyen L., Charu V. (2023). Environmental Pollutants Are Associated With Irritable Bowel Syndrome in a Commercially Insured Cohort of California Residents. Clin. Gastroenterol. Hepatol..

[157] Soares A., Guieysse B., Jefferson B., Cartmell E., Lester J.N. (2008). Nonylphenol in the Environment: A Critical Review on Occurrence, Fate, Toxicity and Treatment in Wastewaters. Environ. Int..

[158] Wagner M., Schlüsener M.P., Ternes T.A., Oehlmann J. (2013). Identification of Putative Steroid Receptor Antagonists in Bottled Water: Combining Bioassays and High-Resolution Mass Spectrometry. PLoS ONE.

[159] Perl D.P., Fogarty U., Harpaz N., Sachar D.B. (2004). Bacterial-Metal Interactions: The Potential Role of Aluminum and Other Trace Elements in the Etiology of Chrohn’s Disease. Inflamm. Bowel Dis..

[160] Jowett S.L. (2004). Influence of Dietary Factors on the Clinical Course of Ulcerative Colitis: A Prospective Cohort Study. Gut.

[161] Aamodt G., Bukholm G., Jahnsen J., Moum B., Vatn M.H., The IBSEN Study Group (2008). The Association Between Water Supply and Inflammatory Bowel Disease Based on a 1990–1993 Cohort Study in Southeastern Norway. Am. J. Epidemiol..

[162] Jädert C., Petersson J., Massena S., Ahl D., Grapensparr L., Holm L., Lundberg J.O., Phillipson M. (2012). Decreased Leukocyte Recruitment by Inorganic Nitrate and Nitrite in Microvascular Inflammation and NSAID-Induced Intestinal Injury. Free Radic. Biol. Med..

[163] Zhou S., Chai P., Dong X., Liang Z., Yang Z., Li J., Teng G., Sun S., Xu M., Zheng Z.-J. (2023). Drinking Water Quality and Inflammatory Bowel Disease: A Prospective Cohort Study. Environ. Sci. Pollut. Res..

[164] Johnson G.J., Cosnes J., Mansfield J.C. (2005). Review Article: Smoking Cessation as Primary Therapy to Modify the Course of Crohn’s Disease. Aliment. Pharmacol. Ther..

[165] Rozich J.J., Holmer A., Singh S. (2020). Effect of Lifestyle Factors on Outcomes in Patients With Inflammatory Bowel Diseases. Am. J. Gastroenterol..

[166] Higuchi L.M., Khalili H., Chan A.T., Richter J.M., Bousvaros A., Fuchs C.S. (2012). A Prospective Study of Cigarette Smoking and the Risk of Inflammatory Bowel Disease in Women. Am. J. Gastroenterol..

[167] Nasr S., Nsiri I., Fredj M.B. (2023). Effectiveness of Smoking Cessation Interventions for Smokers with Crohn’s Disease: A Systematic Review. Future Sci. OA.

[168] Nunes T., Etchevers M.J., Merino O., Gallego S., García-Sánchez V., Marín-Jiménez I., Menchén L., Barreiro-de Acosta M., Bastida G., García S. (2013). High Smoking Cessation Rate in Crohn’s Disease Patients after Physician Advice—The TABACROHN Study. J. Crohns Colitis.

[169] Cosnes J., Beaugerie L., Carbonnel F., Gendre J. (2001). Smoking Cessation and the Course of Crohn’s Disease: An Intervention Study. Gastroenterology.

[170] Sofia M.A., Lipowska A.M., Zmeter N., Perez E., Kavitt R., Rubin D.T. (2020). Poor Sleep Quality in Crohn’s Disease Is Associated With Disease Activity and Risk for Hospitalization or Surgery. Inflamm. Bowel Dis..

[171] Zhang J.-Z., Song X.-Z., Song X.-N., Shen Y.-L., Tang H., Li H. (2024). Prevalence and Risk Factors of Sleep Disorders in Inflammatory Bowel Disease: A Cross-Sectional Study. Int. J. Colorectal Dis..

[172] Ali T., Orr W.C. (2014). Sleep Disturbances and Inflammatory Bowel Disease. Inflamm. Bowel Dis..

[173] Vieujean S., Caron B., Haghnejad V., Jouzeau J.-Y., Netter P., Heba A.-C., Ndiaye N.C., Moulin D., Barreto G., Danese S. (2022). Impact of the Exposome on the Epigenome in Inflammatory Bowel Disease Patients and Animal Models. Int. J. Mol. Sci..

[174] Dougherty U., Mustafi R., Zhu H., Zhu X., Deb D., Meredith S.C., Ayaloglu-Butun F., Fletcher M., Sanchez A., Pekow J. (2021). Upregulation of Polycistronic microRNA-143 and microRNA-145 in Colonocytes Suppresses Colitis and Inflammation-Associated Colon Cancer. Epigenetics.

[175] Wei M., Gao X., Liu L., Li Z., Wan Z., Dong Y., Chen X., Niu Y., Zhang J., Yang G. (2020). Visceral Adipose Tissue Derived Exosomes Exacerbate Colitis Severity via Pro-Inflammatory MiRNAs in High Fat Diet Fed Mice. ACS Nano.

[176] Timms V.J., Daskalopoulos G., Mitchell H.M., Neilan B.A. (2016). The Association of Mycobacterium Avium Subsp. Paratuberculosis with Inflammatory Bowel Disease. PLoS ONE.

[177] Guo Z., Cai X., Guo X., Xu Y., Gong J., Li Y., Zhu W. (2018). Let-7b Ameliorates Crohn’s Disease-Associated Adherent-Invasive E Coli Induced Intestinal Inflammation via Modulating Toll-Like Receptor 4 Expression in Intestinal Epithelial Cells. Biochem. Pharmacol..

[178] Melhem H., Hansmannel F., Bressenot A., Battaglia-Hsu S.-F., Billioud V., Alberto J.M., Gueant J.L., Peyrin-Biroulet L. (2016). Methyl-Deficient Diet Promotes Colitis and SIRT1-Mediated Endoplasmic Reticulum Stress. Gut.

[179] Trakman G.L., Lin W.Y.Y., Hamilton A.L., Wilson-O’Brien A.L., Stanley A., Ching J.Y., Yu J., Mak J.W.Y., Sun Y., Niu J. (2022). Processed Food as a Risk Factor for the Development and Perpetuation of Crohn’s Disease—The ENIGMA Study. Nutrients.

[180] De Chambrun G.P., Body-Malapel M., Frey-Wagner I., Djouina M., Deknuydt F., Atrott K., Esquerre N., Altare F., Neut C., Arrieta M.C. (2014). Aluminum Enhances Inflammation and Decreases Mucosal Healing in Experimental Colitis in Mice. Mucosal Immunol..

[181] Becker H.M., Bertschinger M.M., Rogler G. (2012). Microparticles and Their Impact on Intestinal Immunity. Dig. Dis..

[182] Rappaport S.M. (2011). Implications of the Exposome for Exposure Science. J. Expo. Sci. Environ. Epidemiol..

[183] Vel Szic K.S., Declerck K., Vidaković M., Vanden Berghe W. (2015). From Inflammaging to Healthy Aging by Dietary Lifestyle Choices: Is Epigenetics the Key to Personalized Nutrition?. Clin. Epigenet..

[184] Lippai D., Bala S., Catalano D., Kodys K., Szabo G. (2014). Micro-RNA-155 Deficiency Prevents Alcohol-Induced Serum Endotoxin Increase and Small Bowel Inflammation in Mice. Alcohol. Clin. Exp. Res..

[185] Lv Q., Xing Y., Liu J., Dong D., Liu Y., Qiao H., Zhang Y., Hu L. (2021). Lonicerin Targets EZH2 to Alleviate Ulcerative Colitis by Autophagy-Mediated NLRP3 Inflammasome Inactivation. Acta Pharm. Sin. B.

[186] Galleggiante V., De Santis S., Liso M., Verna G., Sommella E., Mastronardi M., Campiglia P., Chieppa M., Serino G. (2019). Quercetin-Induced miR-369-3p Suppresses Chronic Inflammatory Response Targeting C/EBP-β. Mol. Nutr. Food Res..

[187] James S., Aparna J.S., Babu A., Paul A.M., Lankadasari M.B., Athira S.R., Kumar S.S., Vijayan Y., Namitha N.N., Mohammed S. (2021). Cardamonin Attenuates Experimental Colitis and Associated Colorectal Cancer. Biomolecules.

[188] Qiao C.-X., Xu S., Wang D.-D., Gao S.-Y., Zhao S.-F., Zhang M.-L., Yu B., Yin Q., Zhao G. (2018). MicroRNA-19b Alleviates Lipopolysaccharide-Induced Inflammatory Injury in Human Intestinal Cells by up-Regulation of Runx3. Eur. Rev. Med. Pharmacol. Sci..

[189] Ananthakrishnan A.N., Khalili H., Konijeti G.G., Higuchi L.M., de Silva P., Korzenik J.R., Fuchs C.S., Willett W.C., Richter J.M., Chan A.T. (2013). A Prospective Study of Long-Term Intake of Dietary Fiber and Risk of Crohn’s Disease and Ulcerative Colitis. Gastroenterology.

[190] Hou J.K., Abraham B., El-Serag H. (2011). Dietary Intake and Risk of Developing Inflammatory Bowel Disease: A Systematic Review of the Literature. Am. J. Gastroenterol..

